# Challenges of the infiltration method for halide-based solid‑state battery cathodes

**DOI:** 10.1038/s41598-026-47289-w

**Published:** 2026-04-05

**Authors:** Artur Tron, Alexander Beutl, Andrea Paolella, Pierre Lannelongue, Pedro Lopez-Aranguren

**Affiliations:** 1 AIT Austrian Institute of Technology GmbH, Center for Transport Technologies, Battery Technologies, Giefinggasse 2, Vienna, 1210 Austria; 2 Dipartimento di Scienze Chimiche e Geologiche, Universitàt degli Studi di Modena e Reggio Emilia, Via Campi 103, Modena, 41125 Italy; 3 Center for Cooperative Research on Alternative Energies (CIC energiGUNE), Basque Research and Technology Alliance (BRTA), Parque Tecnológico de Álava, Albert Einstein, 48, Vitoria-Gasteiz, 01510 Spain

**Keywords:** Li_3_YCl_4_Br_2_, Halide solid electrolyte, Infiltration, NCM cathode, All-solid-state battery, Chemistry, Energy science and technology, Engineering, Materials science

## Abstract

Halide solid electrolytes are promising materials for scalable solid-state batteries, but their compatibility with conventional wet-processing methods is still not well understood. In this work, we examine the challenges that arise when a solvent-based infiltration approach is applied to poly crystalline NCM622 cathodes using Li_3_YCl_4_Br_2_ electrolyte solutions. We find that the electrolyte undergoes dissolution and reprecipitation in ethanol and DI water, which significantly lowers its ionic conductivity and leads to the formation of large agglomerates. These solvent-induced structural and morphological changes limit the ability of the electrolyte to penetrate the porous NCM cathode and result in uneven surface coverage. Furthermore, interactions between the solvent, electrolyte, and NCM622 trigger interfacial side-reaction products originating from the partial dissolution of cathode surface species. These reactions increase resistance, disrupt contact between the active material and electrolyte, and ultimately hinder Li-ion transport, causing severe capacity fading. Thus, this study clarifies the fundamental incompatibilities between halide electrolytes and wet-infiltration processing and provides guidance for improving solvent selection, electrode porosity, and interfacial stability in future solid-state battery designs.

**Supplementary Information:**

The online version contains supplementary material available at 10.1038/s41598-026-47289-w.

## Introduction

Solid-state lithium metal batteries (SSBs) have garnered significant attention due to their safety and high energy density^1^. However, ongoing efforts are required to optimize chemistry to develop suitable solid electrolytes (SEs), which are crucial for achieving the desired performance^2,3^. Various chemistries of solid electrolytes have been proposed in the literature, including oxides (e.g., Li_2_O-M_x_O_y_^4,5^, LISICON-^6^, garnet-^7^, and perovskite-^8^, oxynitrides (LIPON-^9^, phosphates (NASICON-^10^, argyrodite-based electrolytes^11^, and halides^12^. These materials are of interest due to their high ionic conductivity (1 mS cm^− 1^) and favorable cycling performance^13,14^. The processability of oxide and phosphate solid electrolytes is constrained not only by their requirement for high-temperature densification but also by factors such as particle-size distribution and mixing quality, which strongly influence interfacial contact with NCM cathodes^15,16^. These considerations are similarly important for sulfide and halide electrolytes, where optimized mechanical mixing can significantly enhance electrode-electrolyte contact without the need for thermal treatment^17,18^. On the contrary sulfide electrolytes exhibit instability in the presence of moisture^19^, potentially limiting cathode/electrolyte compatibility, leading to increased interfacial resistance, high polarization, and reduced specific capacity^20^. Recent studies have demonstrated significant progress in processing Li_6_PS_5_Cl (LPSC) sulfide electrolytes, including advanced dry-processing routes for cathodes, mechanically optimized electrolyte films, emerging LPSC-based chemistries, and extrusion-based fabrication of composite electrodes and separators, highlighting the rapid evolution of both dry and wet chemical approaches^21–27^.

Recently, a new class of solid electrolytes based on halides (Li_3_MX_6_, where M = Y or In; X = Cl, Br, or I) has been explored, demonstrating several advantages over sulfides and attracting rapidly growing research interest^28,29^. Sulfide and halide electrolytes do not inherently share wide electrochemical stability windows, instead, both exhibit chemistry‑specific reactivity and can undergo interfacial decomposition with high-voltage oxide cathodes. Their practical stability is therefore determined by processing conditions, particle‑size control, and the quality of the cathode-electrolyte interface. Halide materials such as Li_3_YCl_6_ and Li_3_YBr_6_ are particularly noteworthy due to their high room‑temperature ionic conductivity (1 mS cm^−1^), high cathodic potential, and robust mechanical stability of the solid-solid ionic contact. In addition, they do not release hazardous H_2_S gas and can be handled in dry rooms with relatively high dew points, making them attractive and practical for safe, high-energy-density solid-state batteries^30^. Beyond these attributes, halide‑based solid electrolytes also benefit from improved oxidative stability compared to sulfides, generally lower grain-boundary resistance than many oxide electrolytes, and good processability for scalable manufacturing. Although halide electrolytes offer attractive stability and compatibility with high-voltage cathodes, their use is limited by the high cost of elements such as yttrium and indium, and by challenges associated with wet‑chemical processing, including solvent‑induced side reactions and agglomeration^25,31^. These factors must be weighed alongside their advantages when considering halides as candidates for infiltration-based solid-state battery architectures.

Dry processing routes for electrolyte and cathode films present challenges related to mixing uniformity and interfacial contact, yet they also offer notable advantages, including scalability, solvent-free fabrication, reduced contamination, and good mechanical properties when particle size and pressing conditions are optimized, making them an increasingly promising approach for large-scale production^12,24,25,32^. Consequently, the wet chemical process remains more scalable for producing small and large cathode, anode, and electrolyte films. However, as previously reported^33–35^, wet-chemical films suffer from issues related to workability and low electrochemical performance, which require optimization. Controlling side reactions is essential for fabricating electrode and electrolyte layers, but the range of solvents and binders compatible with halide electrolytes is limited compared to the standard PVDF binder and NMP solvent used in lithium-ion batteries^35,36^.

About sulfide electrolytes, the infiltration route typically involves preparing a dispersion of ceramics in ethanol (EtOH) solvent, which is then combined with standard PVDF-based porous electrodes used in lithium-ion batteries. This method offers a valuable alternative to wet chemistry film processing. Studies^37–40^ have reported significant progress in infiltrating solid sulfide electrolytes into conventional lithium-ion NCM cathode and Si anode chemistries based on the PVDF/NMP system, maintaining electrochemical performance comparable to that of NCM cathodes and Si anodes in lithium-ion batteries. However, our group have previously highlighted the challenges of the infiltration process, such as issues related to the different pore sizes of conventional NCM811 electrodes and the particle size of Li_6_PS_5_Cl electrolyte solution in EtOH. These challenges result in poor specific capacities and high resistance due to the formation of a sulfide layer on the cathode surface and unwanted side reactions between sulfide and PVDF/NMP^41^. Therefore, the infiltration process of sulfide electrolyte solutions for conventional cathode and anode materials based on the PVDF/NMP system is either not feasible or requires optimization to maintain stable electrochemical performance. Table 1 summarizes the best electrochemical performances of the infiltration method reported in the literature. It is important to note that the infiltration method aims to: (a) minimize the use of ceramic electrolytes while maintaining high electrochemical performance, (b) reduce the amount of toxic sulfide electrolytes and associated costs, and (c) increase overall energy density. In order to achieve these goals, it is crucial to dissolve or disperse sulfide electrolytes well in ethanol to fill as many pores as possible using minimal sulfide electrolyte solution. However, the literature on the infiltration process of halide-based solid electrolytes into conventional PVDF/NMP-based cathode materials remains limited or lacking.

**Table 1:** Reported data on the feasibility of infiltration route for composite electrode processing (*after dissolution/ reprecipitation).

Active material	Infiltrated solid electrolyte	Solvent	Post-treatment	Ionic conductivity after dissolution, S cm^− 1^*	Specific capacity, mAh g^− 1^	References
LiCoO_2_/PVDF/NMP/Super P Graphite/PVDF/NMP/Super P	Li_6_PS_5_Cl, 0.4LI-0.6Li_4_SnS_4_	Ethanol, Methanol	Heating up to 180 °C under vacuum	1.9 10^− 4^	141 (at 0.14 mA cm^− 2^) 364 (at 0.14 mA cm^− 2^)	^37^ ^37^
Silicon/ PVDF/NMP/Super P	Li_6_PS_5_Cl	Ethanol	Heating up to 180 °C under vacuum	10^− 4^	3000 (at 0.25 mA cm^− 2^)	^38^
NMC622/PVDF/NMP/Super P	Li_6_PS_5_Cl	Ethanol	Heating up to 180 °C under vacuum	10^− 4^	136 (at 0.05 C)	^39^
NMC811/PVDF/NMP/Super P	Li_6_PS_5_Cl	Ethanol	Heating up to 180 °C under vacuum	10^− 4^	196 (at 0.1 C)	^40^
NMC811/PVDF/NMP/Super P	Li_6_PS_5_Cl	Ethanol	Heating up to 180 °C under vacuum	10^− 4^	Less than 1 (at 0.05 C)	^41^

In this study, we demonstrate for the first time that the infiltration of halide nanoparticles into conventional cathodes, when dispersed in ethanol (or/and other solvents such as acetonitrile (ACN), aniline (ANI), dibromomethane (DBM), ethanol (EtOH), hexane (HE), hexyl isobutyrate (HIB), toluene (TOL), xylene (XYL), and deionized (DI) water), results in the formation of micron-scale platelets on the surface. The formation of sub-micron crystals can impact the infiltration process and restrict the diffusion of halides through the tortuous pores of LiNbO_3_-coated LiNi_0.6_Co_0.2_Mn_0.2_O_2_ (NCM622-LiNbO_3_) electrodes. We investigated all relevant parameters, including the chemical nature of the solvent and the operating temperature, related to electrolyte infiltration. Additionally, we focused on the properties of the PVDF-based electrode, its porosity, and the method of infiltrating the halide electrolyte Li_3_YCl_4_Br_2_ (LYCB) to ensure thorough filling of the cathode depth, thereby maximizing ionic and electrical contact. However, the fabrication process using simple infiltration methods, applied to electrodes of conventional lithium-ion batteries (LIBs) with homogeneous solid electrolyte solutions, remains challenging. Several parameters must be controlled: (a) distribution of halide electrolyte particles after drying, (b) chemical compatibility of halide electrolytes with binders, and (c) the infiltration route and resulting electrochemical performance.

## Results and discussion

### Design of infiltration process

**Fig. 1:**
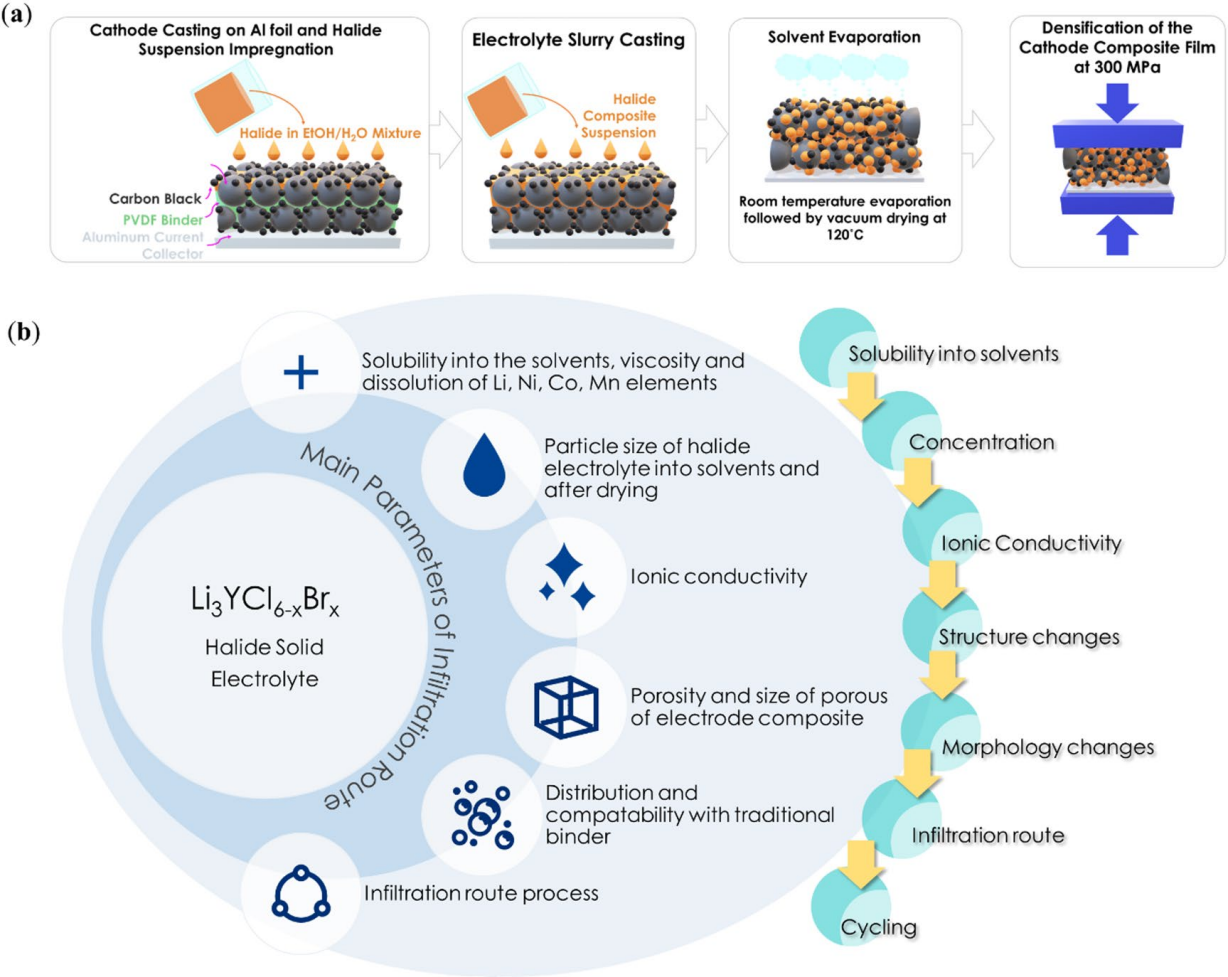
(**a**) Schematic illustration of the solution-based infiltration process of halide-based solid-electrolyte (halide in ethanol solvent) into conventional bare PC-NCM622 based composite cathode, and (**b**) main parameters of Infiltration route.

The fabrication process for conventional lithium-ion batteries with cathodes based on PVDF/NMP system by using simple infiltration methods with homogeneous solid electrolyte solutions must adhere to several critical requirements and controls (Fig. 1): (1) ensuring compatibility between the halide solid electrolyte and solvents, (2) determining the optimal concentration of the halide electrolyte in the solvent, (3) assessing the impact of solvent treatment on ionic conductivity, (4) evaluating structural and morphological changes after treatment, and (5) optimizing the infiltration route process to achieve the desired electrochemical performance.

### Halide solid electrolyte of Li_3_YCl_4_Br_2_—solvent compatibility

**Fig. 2:**
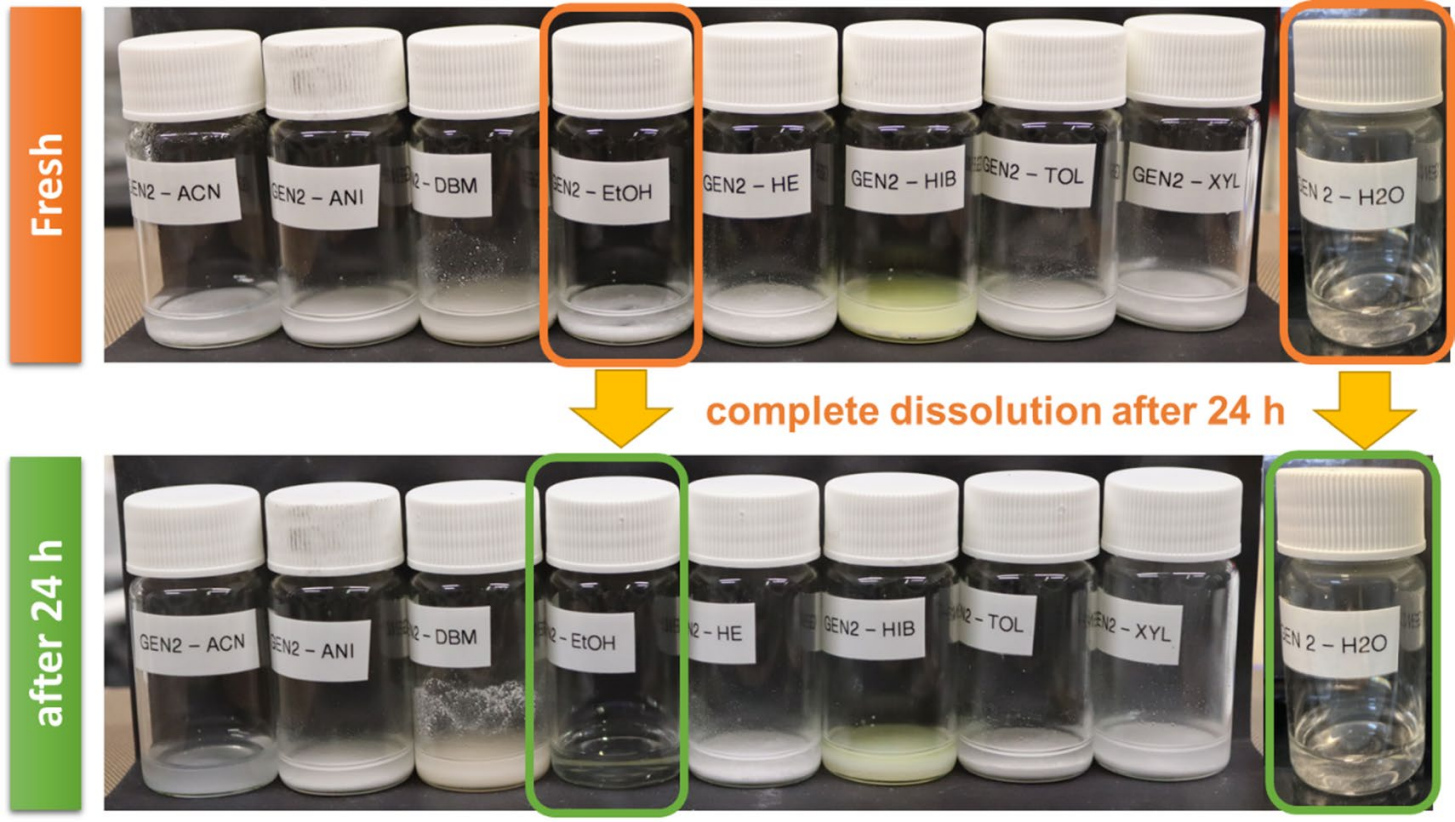
Photographs of vials with Li_3_YCl_4_Br_2_ treated with the solvents ACN, ANI, DBM, EtOH, HE, HIB, TOL, XYL, and DI water with a loading of 10 wt% Li_3_YCl_4_Br_2_ before and after 24 h treatment. All solvents except EtOH and DI water result in the formation of a suspension, while EtOH and DI water dissolve Li_3_YCl_4_Br_2_. The split images of DBM, HIB show the color changes of the mixture due to the side reaction formation, while ACN, ANI, HE, TOL, XYL show the settling of the particles after 24 h related to the migration (sedimentation) of particles.

In order to investigate the effects of various solvents on the halide electrolyte, common solvents such as ACN, ANI, DBM, EtOH, HE, HIB, TOL, XYL, and DI water were employed for Li_3_YCl_4_Br_2_ dispersions. All solvents, except EtOH and DI water, resulted in the formation of a suspension, whereas EtOH and DI water as Lewis bases dissolved Li_3_YCl_4_Br_2_ (Figure S1, Supporting Information). It should be noted that the pH values were assessed using pH indicator paper for the DI water solutions and remained near neutral (6–7). pH could not be determined for ethanol-based systems, as pH is not defined in non-aqueous solvents^42,43^. The dissolution behavior is therefore more appropriately attributed to the Lewis basicity and donor ability of EtOH and H_2_O, which can coordinate Li^+^ and partially dissolve Li_3_YCl_4_Br_2_^44–46^. The split images of DBM and HIB exhibited color changes in the mixture, as result of chemical degradation. In contrast, ACN, ANI, HE, TOL, and XYL showed particle settling after 24 h, attributed to the migration (sedimentation) of particles, with no evident color changes, as illustrated in Fig. 2.

To characterize these degradations, the solvents were removed, and the resulting precipitates were collected for ionic conductivity, pellet density (cf. Supporting Information), XRD, and SEM/EDS analyses. Only the electrolyte precipitates obtained from EtOH, TOL, XYL and DI water dispersions were selected for characterization, as these solvents were estimated to be most promising for further infiltration. In addition, based on the initial solvent screening shown in Fig. 2, EtOH, TOL, XYL, and DI water were selected for detailed characterization. EtOH and DI water were the only solvents that partially dissolved Li_3_YCl_4_Br_2_, enabling the collection of precipitates for post‑mortem analysis. In contrast, TOL and XYL did not dissolve the electrolyte but formed stable, homogeneous dispersions with good wetting of the NCM622 surface, which is essential for evaluating infiltration behavior. The remaining solvents exhibited poor wetting, rapid sedimentation, visible side‑reaction products, or no measurable deposition after 24 h, making them unsuitable for further analysis.

**Fig. 3:**
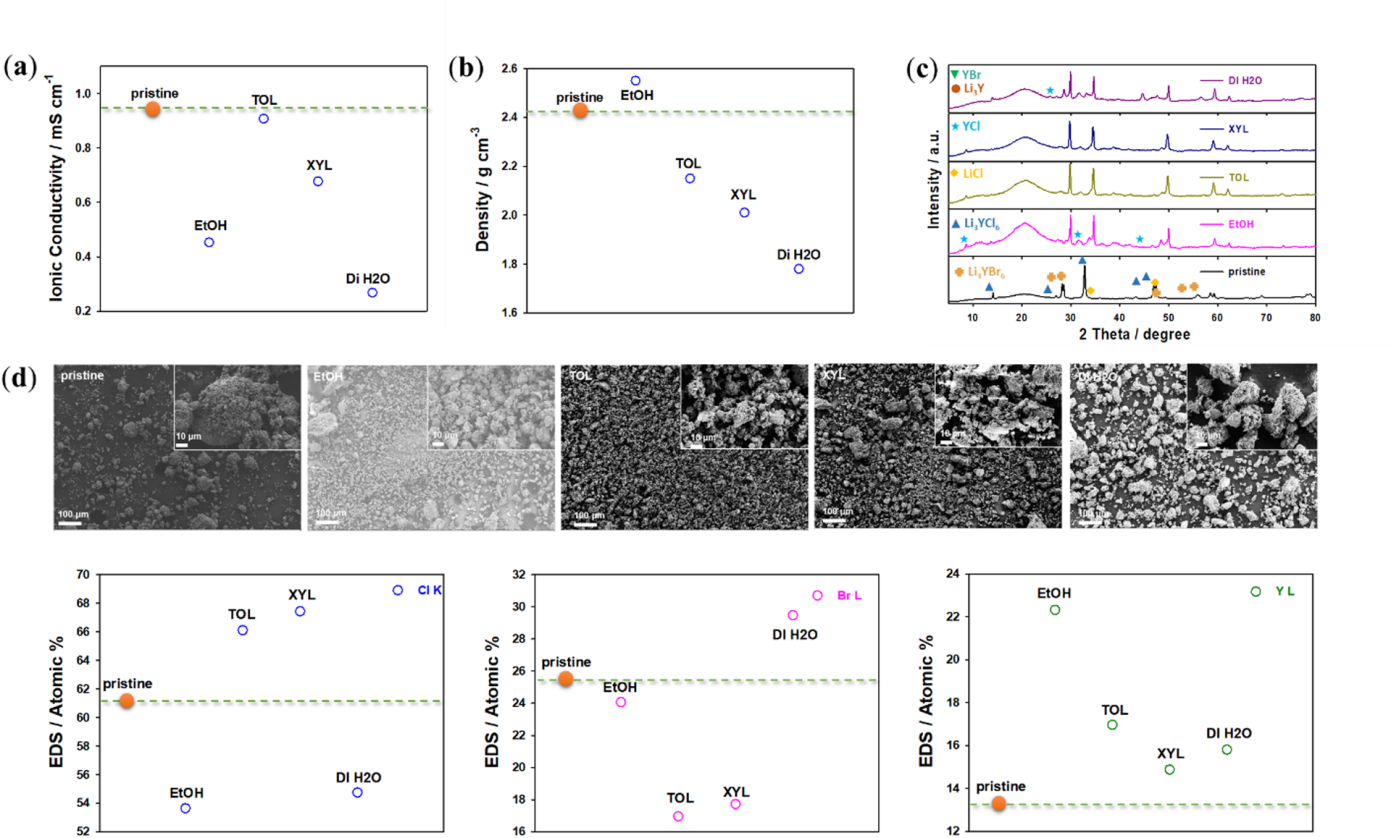
(**a**) Ionic conductivity, (**b**) density and (**c**) XRD pattern and (**d**) SEM images and EDS analysis of Li_3_YCl_4_Br_2_: pristine and treated with the solvents EtOH, TOL, XYL, and DI water after drying at 150 °C in vacuum.

For the DI water-treated sample, the ionic conductivity decreased by approximately one order of magnitude after solvent treatment and drying at 150 °C, consistent with the formation of new impurity phases (Fig. 3a). These impurities, primarily located on the surface of the electrolyte particles, led to high interfacial resistances (grain boundary resistance due to reduced particle-particle contact area), as shown in Fig. 3c. The DI water (2.64 × 10⁻⁴ S cm⁻¹) and EtOH (4.22 × 10^−4^ S cm⁻¹) treatments impacted the ionic conductivity of the solid electrolyte due to the formation of side products, which increased grain boundary resistance (Fig. 3a and Table S1, Supporting Information). These side reaction components and/or decomposed Li_3_YCl_4_Br_2_ electrolytes are either poor conductors or insulative, contributing to increased electrolyte resistance. Similar observations have been reported for halide solid electrolytes after solvent treatment [36]. The DI water-treated sample shows a pronounced decrease in ionic conductivity after drying at 150 °C, dropping by approximately one order of magnitude compared to the pristine Li_3_YCl_4_Br_2_ (Fig. 3a). The XRD analysis reveals the disappearance of characteristic reflections of the parent halide and the emergence of new peaks associated with Y-rich reduced phases (Fig. 3c). While the ICP measurements confirm significant Li and transition-metal leaching (discussed more details below, Fig. 8b). Taken together, these observations indicate the formation of electronically conductive but ionically resistive side‑reaction products. This evidence is attributed to the formation of Li_3_Y-type reduced phases, which are reported to exhibit high electronic conductivity but low ionic conductivity, in contrast to the highly ionically conductive layers formed by YCl, YBr, LiCl, and LiBr components^12,36,47–50^. We note that this interpretation is based on indirect structural and compositional evidence, as dedicated electronic conductivity measurements were not performed in this study. In contrast, the conductivities of precipitates from TOL and XYL, measured at 8.49 × 10⁻⁴ and 5.94 × 10⁻⁴ S cm⁻¹, respectively, retained almost all the pristine conductivity of 8.38 × 10⁻⁴ S cm⁻¹.

For the DI water-treated Li_3_YCl_4_Br_2_ powder, the pellet density after pressing at 300 MPa and drying at 150 °C was lower than that of the pristine sample, consistent with the formation of impurity phases and structural degradation (discussed in more detail below, see Fig. 3c). The lower density (Fig. 3b) is attributed to changes in surface morphology following solvent treatment as observed by SEM.

The XRD patterns of the as-prepared powders match the halide phase as shown in Fig. 3c. After annealing at 150 °C some impurity phases are visible as additional peaks in the diffraction patterns (YCl, YBr, LiCl, LiBr, Li_3_Y). After heating at 150 °C, the intensity of the peaks increased due to higher crystallinity and larger crystallite sizes, with some impurity phases being consumed. These side reaction products likely arise from the hydrolytic decomposition of the halide solid electrolyte particles and can form an insulating passivation layer, which obstructs lithium-ion diffusion at the cathode-solid electrolyte interface. These findings suggest that heat treatment is necessary to further reduce impurities and recover the halide phase. However, due to the thermal stability limits of the cathode, heat treatment above 500 °C can catalyze side reactions such as cation exchange reactions (metal oxide/metal halide exchange).

After solvent treatment, small nanoparticles and some micron-sized crystals with agglomeration and changes in their surface morphologies were observed, resulting in lower ionic conductivity compared to the pristine materials, as shown in Fig. 3d. This phenomenon could be related to Ostwald ripening which is associated with the agglomeration of halide solid electrolytes after solvent treatments^51–53^. EDS analysis of the samples revealed changes in the structure chemistry and the ratio of the components of the halide phase for all solvent-treated samples, along with the possible formation of side reaction components (Fig. 3d and Figure S2, Supporting Information). Especially the Y : X (X = Cl, Br) ratio is reduced for all samples. This indicates a loss of halides, presumably during the drying step by the formation of evaporation of volatile compounds such as HCl, HBr. These changes may explain the lower ionic conductivities and pellet densities observed compared to the original sample.

### Concentration of Halide solid electrolyte of Li_3_YCl_4_Br_2_—solvent impact

**Fig. 4:**
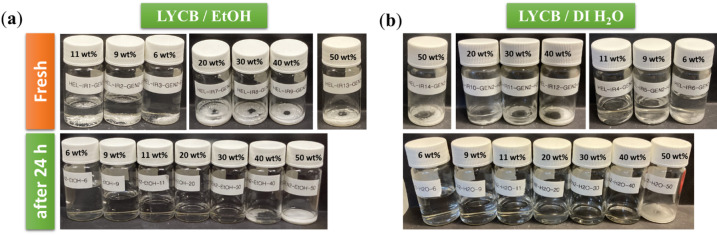
Photographs of vials with Li_3_YCl_4_Br_2_ treated with the solvents (**a**) EtOH and (**b**) DI water with a loading of 50, 40, 30, 20, 11, 9, 6 wt% of Li_3_YCl_4_Br_2_ before and after 24 h treatment.

For the infiltration process solvents which fully dissolve the halide electrolytes are considered most promising. Therefore, based on the obtained results, only EtOH and DI water fully dissolve the electrolyte and produce homogeneous solutions (Fig. 2), whereas the other solvents form only coarse dispersions. However, dissolution does not imply practical suitability, as EtOH and DI water also induce electrolyte degradation. Solid electrolyte solutions were prepared by mixing Li_3_YCl_4_Br_2_ electrolyte with EtOH and DI water, resulting in a transparent dispersion, as shown in Fig. 4. As the concentration of the solid electrolyte solutions decreased from 50 wt% to 6 wt%, the color of the solutions remained consistent, despite variations in the viscosity of the concentrated solutions. (Figure S3, Supporting Information). However, the maximum concentration achievable was 30 wt% for EtOH and 40 wt% for DI water.

**Fig. 5:**
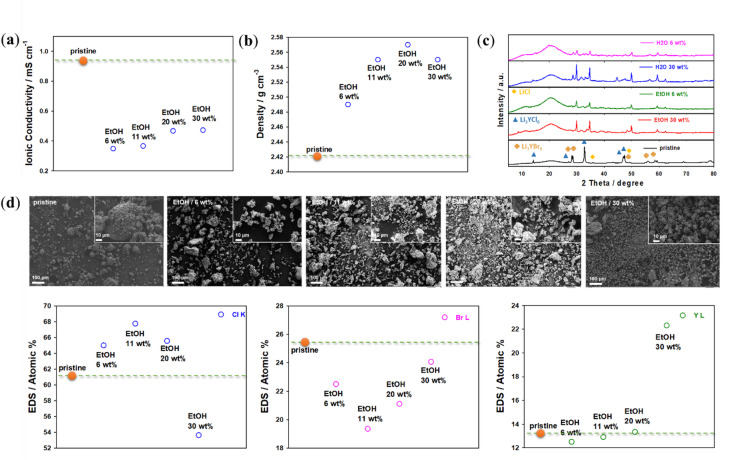
(**a**) Ionic conductivity, (**b**) density and (**c**) XRD pattern and (**d**) SEM images and EDS analysis of Li_3_YCl_4_Br_2_ treated with the solvents EtOH with a loading of 6, 11, 20 and 30 wt% after drying at 150 °C in vacuum.

The ionic conductivity of solid electrolytes is a key parameter governing the performance of solid-state batteries, as higher conductivity and lower particle-to-particle resistance directly enhance cell efficiency. This influence is amplified by the increased conductivity and reduced particle-particle resistance. Figure 5a illustrates the ionic conductivity of the Li_3_YCl_4_Br_2_ solid electrolyte obtained after solvent evaporation from EtOH solutions with varying initial concentrations. All measurements were performed on the dried solid electrolyte powders, not on the liquid solutions (cf. Supporting Information). The data show that increasing the concentration of the halide electrolyte in EtOH solutions leads to an increase in ionic conductivity. This trend suggests that the Li_3_YCl_4_Br_2_ electrolyte enhances Li-ion transport within the solution, attributed to its high viscosity and the substantial presence of Li ions, thereby improving ionic mobility (Figure S3, Supporting Information). Furthermore, samples with lower concentrations of the halide electrolyte in the EtOH solutions exhibited reduced ionic conductivity. This reduction is likely attributed to decreased ionic mobility and a lower dielectric constant, a phenomenon also observed in both conventional non-aqueous and aqueous electrolytes^54^. Furthermore, increasing the halide electrolyte concentration decreases the ratio between residual water of the solvent and halide electrolyte. Thus, relative to the total amount of electrolyte, less side reactions can occur.

It should be noted that the density of the obtained samples with various concentrations is comparable to that of the pristine sample. However, structural and morphological changes occur in the halide powder after solvent treatment, as shown in Fig. 5c and d. The structural changes of Li_3_YCl_4_Br_2_ treated with EtOH at loadings of 6, 11, 20, and 30 wt% were identified by XRD as shown in Fig. 5c. The XRD patterns for the halide electrolytes indicate that the materials crystallize in two distinct phases, depending on the Cl to Br ratio. The Li_3_YCl_6_ sample crystallized in the trigonal P3m1^49,54^ space group, similar to Li_3_YBr_x_Cl_6−x_ with x = 2. However, materials with x > 2, as well as the full bromide Li_3_YBr_6_, crystallized in the monoclinic C2/m^50,55^ space group. The peak positions of the XRD patterns for the halide treated with EtOH and DI water solvents differ from those of pristine Li_3_YCl_4_Br_2_, observed as small shifted peaks. This shift may indicate the impurity formation depending on the concentration of the halide electrolyte. These results demonstrate that solvent-treated samples exhibit changes in the structure and powder morphology of the halide electrolyte powders. It should be noted that the post-mortem XRD analysis revealed only minor peak‑broadening and intensity variations without measurable peak‑position shifts, suggesting surface‑level side reactions and electrolyte crystallization rather than bulk structural degradation (Tables S1 and S4, Supporting Information). Figure 5d clearly shows that after solvent treatment, the halide samples have different surface particles compared to the pristine samples, with the formation of agglomerated particles. Additionally, EDS analysis was performed to confirm the changes in the elemental composition of the halide electrolyte after EtOH and DI water treatment (Fig. 5d and Figure S4, Supporting Information). The EDS spectra indicated the presence of elements different to the nominal ratio expected for Li_3_YCl_4_Br_2_, confirming a change in the halide phase. Furthermore, the concentrations of these elements increased with increasing amounts of halide electrolyte from 6, 11, 20, and 30 wt% for EtOH, with a similar effect observed for DI water, as presented in Figure S4 (Supporting Information) Based on the analyses of ionic conductivity, density, viscosity, XRD, SEM, and EDS, halide electrolyte samples treated with solvents were successfully obtained (Table S4, Supporting Information), with reduced ionic conductivity, though. Both EtOH and DI could be used for the infiltration process of halide electrolyte solutions, facilitating their application in conventional NCM622 cathodes.

The antagonistic relation between viscosity and halide electrolyte concentration should be highlighted, as this has a major impact on the achievable electrolyte saturation levels after infiltration. During the infiltration process, the infiltration solution needs to fill the entire pore volume of the electrodes. The subsequent drying step leads to precipitation of the halide electrolyte and evaporation of the used solvent. Thus, a high electrolyte concentration of the infiltration solution is favorable to minimize the amount of evaporated solvent and thus maximize the saturation level. The evaporated solvents will reduce the saturation level and leave only partially filled pores behind. Although, a high electrolyte concentration of the infiltration solutions is promising in this regard, the increased viscosity, though, will have an inhibiting effect on the saturation level. Higher viscosities of the infiltration solutions impede the filling step as smaller pores are less likely to be filled compared to a low viscosity solution. Thus, non- or only partially filled pores will be left with low amounts of electrolyte. A proper balance needs to be found in order to achieve the best performance.

### Particle size distribution of Halide solid electrolyte of Li_3_YCl_4_Br_2_ – solvent solution – PC-NCM cathode

The PC-NCM622 (poly crystalline, denoted as PC) electrodes with controlled open porosity were fabricated using a conventional PVDF/NMP slurry-casting method. These electrodes were infiltrated with dissolved or well-dispersed Li_3_YCl_4_Br_2_ electrolyte, using 30 vol% SE relative to the cathode. The volume of infiltration solution was selected to match the accessible pore volume of the electrode, ensuring complete pore filling without oversaturation. The detailed porosity-based calculation is provided in the Supporting Information. After infiltration, the electrodes were calendered to reduce porosity to below 70%, which was identified as the minimum threshold that still allows the solid electrolyte to penetrate the deeper electrode regions. In addition, porosity levels significantly below this value hinder electrolyte infiltration, disrupt ionic pathways, and may lead to capacity fading^41^. Key parameters, including solvent type, SE content, and particle size distribution, were optimized to achieve a uniform SE distribution and an effective Li-ion percolation network while minimizing electrolyte usage.

**Fig. 6:**
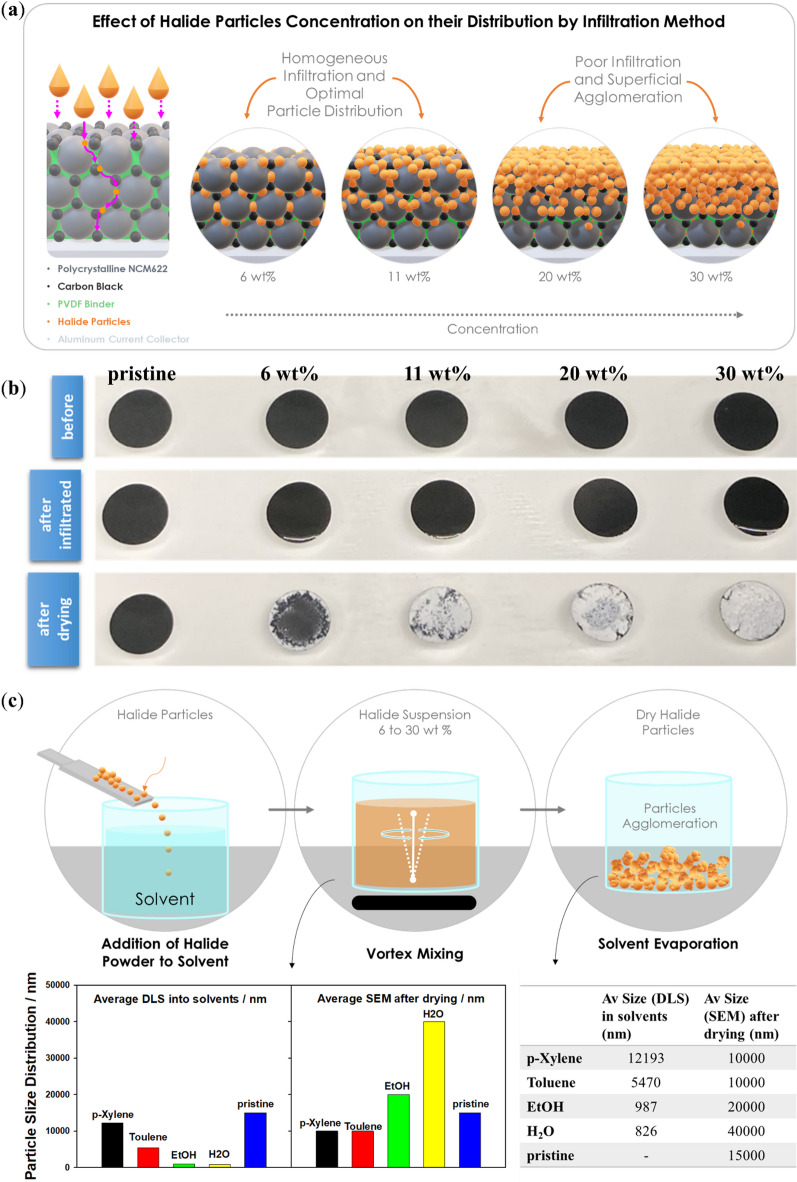
(**a**) Schematic illustration of effect of halide particles concentration on their distribution by infiltration method, (**b**) Photographs of conventional PC-NCM622 cathodes (CAM: CA: Binder / PVDF + NMP) before and after infiltration process via Li_3_YCl_4_Br_2_ into the solvents EtOH with a loading of 0 (i.e. pristine), 6, 11, 20 and 30 wt% and after drying at 150 °C in vacuum. (**c**) Particle size analyzing of Li_3_YCl_4_Br_2_ electrolyte into the solvents EtOH, TOL, XYL, and DI water obtained from DLS analysis and SEM images (Fig. 3).

**Fig. 7:**
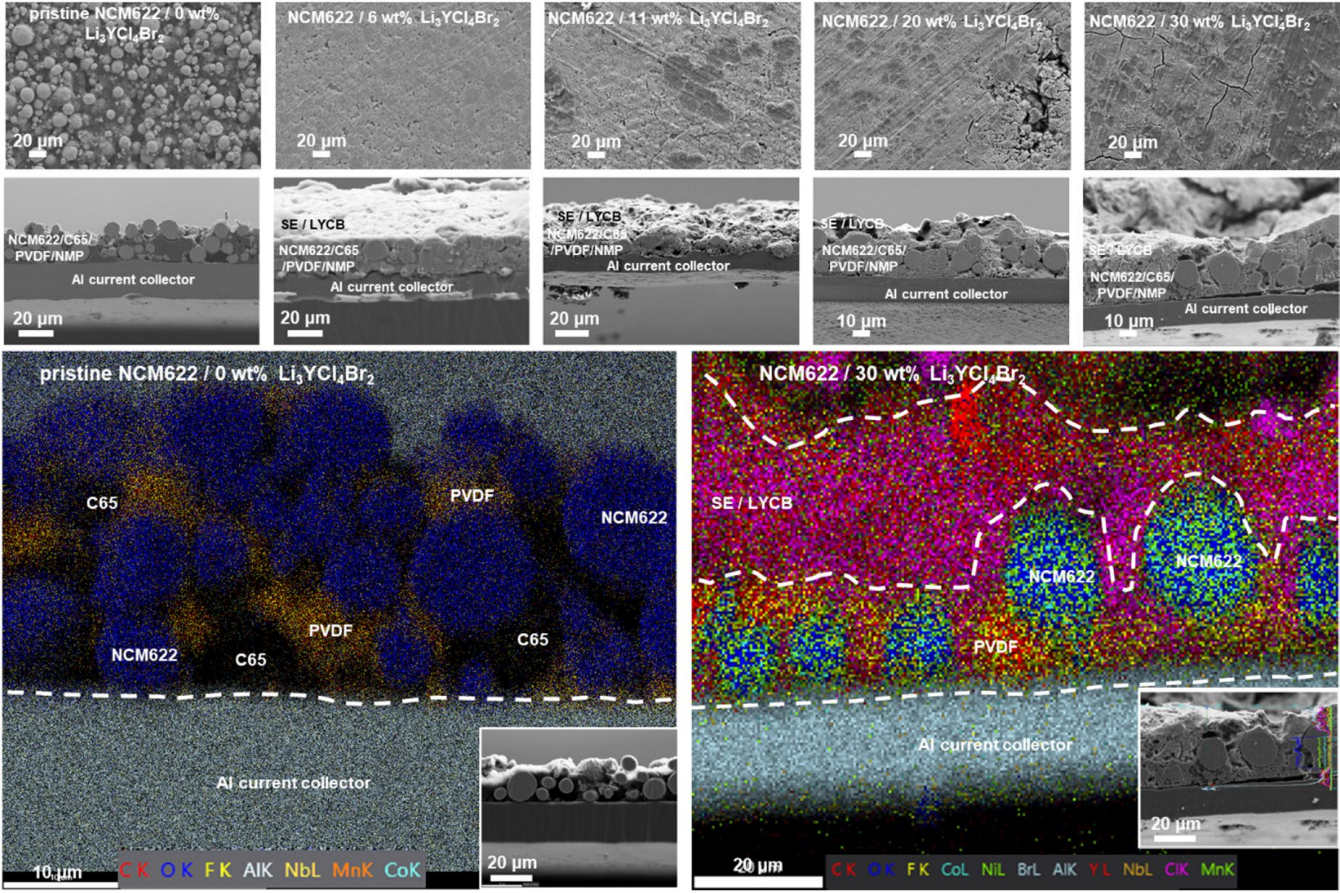
Cross-section of infiltrated PC-NCM622 cathodes via solution Li_3_YCl_4_Br_2_ into EtOH.

As illustrated in Fig. 6a and b, the effect of halide particle concentration (0, 6, 11, 20, and 30 wt%) on their distribution via the infiltration method into NCM622 electrodes shows that most of the electrolyte crystallized on the electrode surfaces rather than infiltrating the pores. The NCM electrodes infiltrated with solid electrolytes before drying exhibited a uniform distribution of Li_3_YCl_4_Br_2_ electrolyte elements. After EtOH treatment, the solid electrolyte was found to accumulate predominantly on the electrode surface, forming large plate-like Li_3_YCl_4_Br_2_ crystals (Fig. 6b). This behaviour is attributed to the complete dissolution of the electrolyte in EtOH and the subsequent migration of dissolved species toward the evaporating surface during drying. The relatively fast evaporation of EtOH at 60 °C promotes this solute redistribution, while slower drying tests still resulted in surface crystallization, indicating that solvent-solute interactions dominate over drying kinetics. Differences in surface energies between NCM622 and the recrystallizing halide electrolyte further favour nucleation at the surface rather than within the pore network. A direct EtOH‑based slurry of NCM, carbon black, PVDF, and Li_3_YCl_4_Br_2_ were not attempted, as EtOH induces electrolyte degradation and would likely exacerbate decomposition during slurry processing. It is important to note that Li_3_YCl_4_Br_2_ solutions in DI water, across concentrations (6, 9, 11, 20, 30, and 40 wt%), exhibited poor wetting on the conventional NCM622 cathodes. The high surface energy of the cathode prevented infiltration into the porous structure, resulting instead in surface retention and crystallization (Figure S5, Supporting Information). In addition to these factors, the drying temperature, drying time, and overall evaporation rate after infiltration also play a decisive role. The evaporation kinetics of ethanol and DI water can lead to rapid surface crystallization before the electrolyte penetrates the porous NCM cathode structure, which may further contribute to the limited infiltration observed in this study. Consequently, EtOH was selected for further investigations. SEM of halide electrolyte particles in EtOH solvent revealed that Li_3_YCl_4_Br_2_ primarily segregates at the electrode surface rather than penetrating the bulk, failing to fill the pores of the NCM622 cathode due to agglomeration (Figs. 6c and 7). During the dissolution of halide electrolyte particles in EtOH, particle sizes were predominantly detected at the electrode surface, with sizes of approximately 1 μm via DLS and 10–20 μm via SEM analysis (Fig. 6c). As shown in Fig. 2, only EtOH and DI water form true solutions of the halide electrolyte, whereas XYL and TOL produce only coarse dispersions. This phenomenon was also observed after 24 h of dissolution in all solvents (XYL, TOL, EtOH, and DI water) (Tables S3 and S4, Supporting Information). The halide electrolyte particles did not dissolve completely, with average particle sizes of approximately 12, 5.5, 0.98, and 0.83 μm for XYL, TOL, EtOH, and DI water, respectively, as determined by DLS (Figure S6, Supporting Information). Although wet ball milling can yield sub‑100 nm suspensions in other material systems, our measurements show that Li_3_YCl_4_Br_2_ does not reach nanoscale dimensions under the tested solvent conditions, even after extended dispersion, due to dissolution‑driven recrystallization and agglomeration. A similar behaviour was observed for the Li_6_PS_5_Cl electrolyte, where particle sizes in XYL and TOL exceeded 1 μm, compared to pristine particles of 10–20 μm (Figure S4, Supporting Information). The pore structure of polycrystalline NCM622 electrodes is dominated by pores in the 10–100 nm range, with occasional larger pores up to several hundred nanometers. Consequently, micrometre-sized particles formed in non-polar solvents cannot infiltrate the electrode architecture, and the formation of large plate-like particles further increases resistance in micropores (Fig. 7). In contrast, when the electrolyte is fully dissolved in EtOH or DI water, infiltration is not limited by pore size, instead, surface-energy differences and solvent-driven solute migration during drying govern the final SE distribution. EtOH and DI water were therefore considered “eligible” only because they fully dissolve the electrolyte and enable complete infiltration, despite inducing degradation. Combining non-polar solvents with high-energy ball milling may offer a promising future route to obtain nanoscale dispersions suitable for infiltration while preserving ionic conductivity.

Thus, the surface morphologies of the halide electrolyte treated with different solvents may depend on the chemical properties of the solvents, such as density, vapor pressure, dielectric constant, and polarity^41^. The initial particles in the solvent contribute to the construction of a bulk monocrystal, which differs in shape from the primary particles. Figure 6b illustrates schematically the Ostwald ripening concept for the halide solid electrolytes after solvent treatments. In this concept, solid electrolyte primary particles form aggregates.

### Electrochemical evaluation of Halide solid electrolyte of Li_3_YCl_4_Br_2_ – solvent solution – NCM cathode

**Fig. 8:**
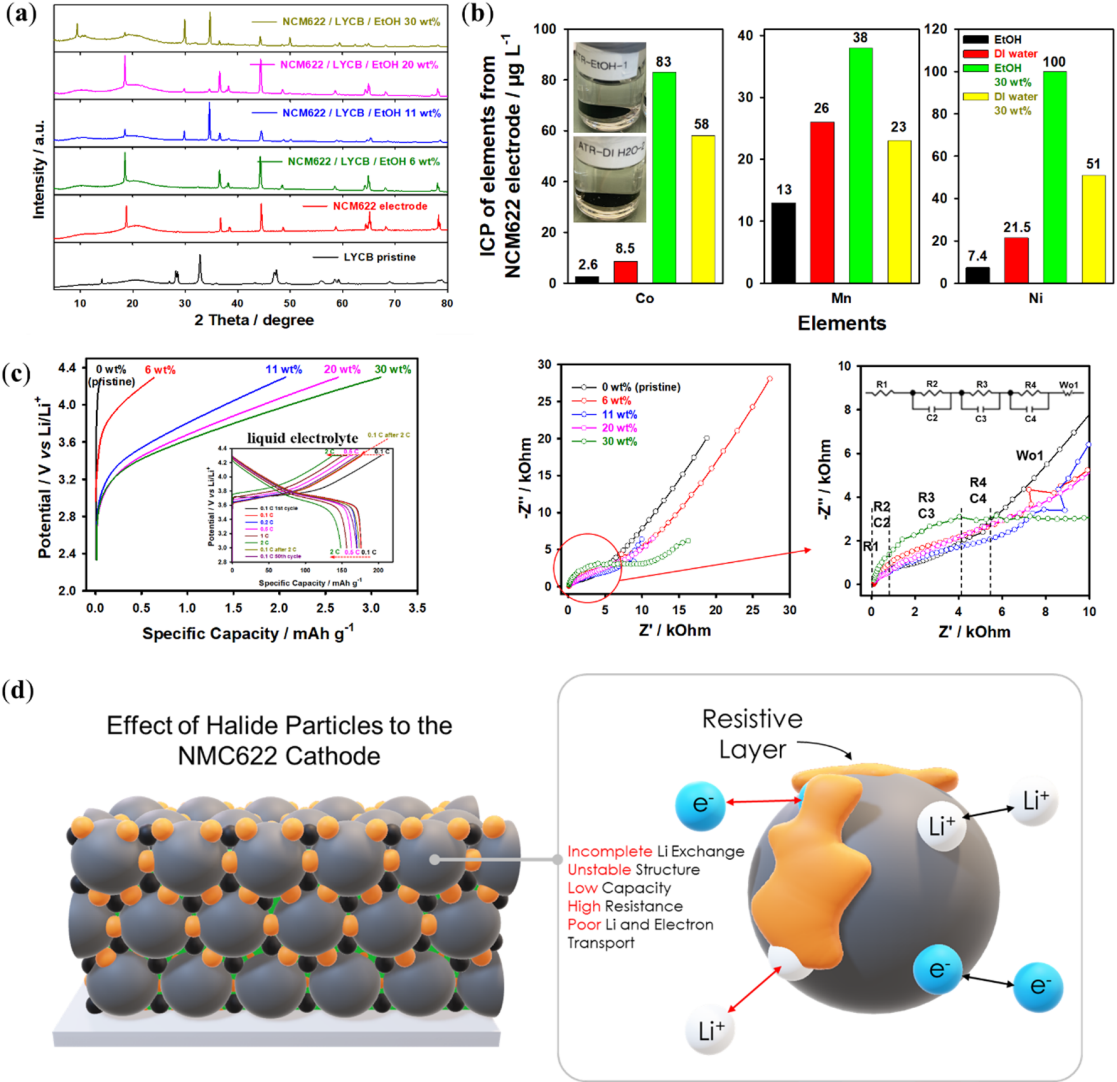
(**a**) XRD pattern, (**b**) ICP of pristine PC-NCM622 cathodes into pure DI H_2_O, pure EtOH, solution of Li_3_YCl_4_Br_2_ into the solvents EtOH and DI H_2_O with a loading of 30 wt%, respectively, (**c**) Cycling data (included liquid electrolyte as reference, see Figure S8, Supporting Information) with Nyquist plots of infiltrated PC-NCM622 cathodes via solution Li_3_YCl_4_Br_2_ (LYCB) into EtOH, and (**d**) Schematic illustration of the degradation mechanism of infiltrated cathodes.

XRD measurements were conducted to identify any changes in the crystal structure of the NMC622 cathode electrode and the Li_3_YCl_4_Br_2_ electrolyte, as shown in Fig. 8a. XRD measurements (Fig. 8a) showed slight modifications in the NCM622 crystalline structure after infiltration with Li_3_YCl_4_Br_2_ electrolyte. With increasing electrolyte content on the electrode surface, the intensity of NCM622 peaks decreased, while the intensity of Li_3_YCl_4_Br_2_ peaks (low‑ and high‑angle reflections) increased, indicating deposition of crystalline electrolyte and partial surface coverage of the cathode. ICP analysis (Fig. 8b) confirmed the occurrence of chemical interactions between the NCM cathode and the electrolyte in EtOH solutions, evidenced by measurable Ni, Mn, Co, and Li concentrations (7.4, 13, 2.6, and 200 µg L⁻¹, respectively). These concentrations were substantially higher in DI water (21, 26, 8.5, and 1100 µg L⁻¹; Figure S7, Supporting Information), demonstrating that while dissolution is reduced in EtOH, it remains significant. Such leaching of transition-metal and lithium species indicates the formation of interfacial side‑reaction products that compromise cathode-electrolyte contact, providing a plausible explanation for the diminished electrochemical performance (Fig. 8c). While Li-In alloys can offer a more stable and reproducible reference interface in sulfide and halide systems^56^, as demonstrated in our previous work^50^, Li metal was intentionally used here to directly probe the behavior relevant to Li-metal solid‑state cells. It should be noted, however, that leaching is not the only factor contributing to the reduced electrochemical performance. Electrolyte deposition on the cathode surface, the relatively high porosity of the infiltrated electrodes, and incomplete interfacial contact is also likely to play significant roles. A toluene-based slurry route may mitigate some of these issues. However, it was not explored in this study and would require nanoscale comminution of the electrolyte to ensure sufficient ionic percolation. Furthermore, the use of Li metal and a single-layer separator may influence the absolute capacity values. More robust configurations employing Li-In alloy anodes and bilayer sulfide/halide separators will be considered in future work to obtain more reliable electrochemical data.

To understand the lithium-ion transport kinetics through NCM622 cathode material and infiltrated Li_3_YCl_4_Br_2_ solid electrolytes, electrochemical impedance spectroscopy (EIS) measurements and specific capacity evaluations were performed (Fig. 8c). The Nyquist plots of the NCM622 electrodes were obtained and fitted using an equivalent circuit, with a detailed discussion on the influence of each element on surface resistivity^11,41,57^. In the equivalent circuit for the obtained samples, resistances are identified as follows: R1 represents the solid electrolyte layer between the cathode (Rc as R2 is path resistance and R3 as interfacial resistance) and anode (Ra as R4 is the interface between the lithium anode and solid electrolyte), along with Warburg (W) impedance. The assignment of each resistance element was based on their characteristic frequency domains in the Nyquist and Bode plots, scaling behaviour with electrolyte thickness (R1) and interfacial area (R3), and sensitivity to infiltration depth and particle size (R2), Kramers-Kronig validation confirmed that the chosen model adequately represents the physical processes in the cells^11,41,50^. It should be noted that the resistances R2 and R3 are limiting factors for charge transfer between the NCM622 cathode and the halide electrolyte due to the lower penetration of the solid electrolyte during the infiltration process. The Li_3_YCl_4_Br_2_ solid electrolyte layer acts as an insulating layer limiting the lithium-ion transport capability (Fig. 8d). Moreover, the ICP measurements revealed measurable leaching of Li and transition-metal species from NCM into both DI water and EtOH, confirming that these solvents partially dissolve Li_3_YCl_4_Br_2_ due to their Lewis basicity. During drying, the dissolved halide species re-crystallize on the CAM surface, producing a discontinuous and resistive Li_3_YCl_4_Br_2_ layer. Such a layer impedes Li^+^ transport across the cathode-electrolyte interface (Fig. 8d), which, together with the surface crystallization and poor pore penetration, explains the limited electrochemical performance of the infiltrated electrodes (Fig. 8c). The resistance of the solid electrolyte (R1) decreases with increasing concentrations of halide electrolyte on the surface of the NCM cathode. Moreover, the increase in path resistance (R2) and the limited pathways of solid electrolytes during the infiltration process into the deeper regions of the cathode composite, due to the large particles compared to the pore size of the NCM622 electrode, lead to poor electrochemical performance compared the NCM622 electrode tested into the conventional liquid electrolyte (Figure S8, Supporting Information) and with these materials into NCM622 / Li_3_YCl_4_Br_2_ solid electrolyte / Li metal anode all-solid-state battery cell^50^. The resistance R4 (Ra) reflects the resistance between the lithium anode and the solid electrolyte, which increases from low to high porosity of the cathode due to the microstructure of the cathode composite and lower contact between the lithium anode and solid electrolyte. Regarding lithium-ion diffusion, the solid electrolyte layer formed on the cathode surface and within the infiltrated solid electrolyte can significantly enhance cyclic stability. However, the formation of large particles causes capacity fading after the first cycle, as this solid electrolyte layer acts as an insulating barrier, hindering lithium-ion transport (Fig. 8d), similar effect observed for sulfide-based solid electrolyte and NCM811/PVDF/NMP-based cathode^41^. Therefore, it can be inferred that the infiltration of the solid electrolyte into conventional cathode electrodes depends on several factors, including the solvent evaporation rate, the surface morphology of the solid electrolyte, and the particle size of the porous cathodes. In addition, the dissolution particle size of the halide electrolyte in different solvents and the surface-energy interactions between the electrolyte, solvent, and cathode framework also play important roles (Table S5, Supporting Information). These properties are of great importance in the fabrication of commercial electrodes based on conventional binders and solvent systems for achieving high capacity, long cycle life, safety, and scalable solid-state batteries with halide and sulfide-based solid electrolytes.

Thus, we can conclude that the formation of side-reaction products at the cathode-electrolyte interface can significantly impair the performance of all-solid-state batteries by disrupting both ionic and electronic pathways. In the present system, chemical interactions between the NCM622 surface and the Li_3_YCl_4_Br_2_ electrolyte during and after infiltration can generate poorly conducting phases such as transition-metal halides or oxyhalides, as well as decomposition products from the binder. These secondary phases act as insulating layers, physically separating the active material from the electrolyte and impeding Li-ion transport. Moreover, volumetric changes associated with the growth of these products can create micro-gaps or voids at the interface, further reducing effective contact area and increasing interfacial resistance. Over extended cycling, this progressive loss of interfacial integrity accelerates capacity fade and degrades rate capability. Analogous degradation phenomena are observed in high-performance structural composites, where interfacial damage between reinforcement and matrix materials compromises load transfer and overall mechanical integrity. As discussed in^58^, mitigating such damage or enabling the interface to recover its functional continuity is key to sustaining system performance. Drawing on this parallel, future work on solid‑state batteries could explore interface-stabilising or self-repairing material approaches to counteract side-reaction-induced contact loss.

In addition to addressing processing and interfacial challenges, optimization of the infiltration process has direct implications for the long-term performance and operational lifespan of all-solid-state batteries. Uniform penetration of the halide electrolyte into the porous NCM622 cathode promotes continuous and intimate contact at the cathode-electrolyte interface, reducing interfacial resistance and mitigating void formation during cycling. These effects help to suppress localized side reactions, thereby slowing capacity fading and improving mechanical integrity over extended operation. Such improvements are particularly relevant for high-demand applications including electric vehicles, grid-scale storage, and aerospace systems where safety, power delivery, and cycle life are critical metrics. The relevance of materials optimization beyond the battery field is exemplified by analogous strategies in other disciplines, such as those reported in^59^, where precise control of material infiltration and interfacial stability enhances long‑term functional performance. Although the application domains differ, the shared principle of interface engineering to extend service life underscores the broader scientific and technological value of our findings.

We can conclude that the results highlight both the potential and the current limitations of solvent‑assisted infiltration for Li_3_YCl_4_Br_2_-NCM622 cathodes. While infiltration enables partial electrolyte incorporation into the porous framework, the resulting cathodes still exhibit limited electrochemical performance due to surface deposition, incomplete percolation, and interfacial instability. Alternative processing routes such as direct slurry-casting of NCM, conductive carbon, binder, and halide electrolyte in non-polar solvents, or nanoscale comminution of LYCB dispersions prior to infiltration may offer more effective pathways to achieve uniform electrolyte distribution without compromising ionic conductivity. These strategies represent important directions for future work aimed at improving halide-based solid-state cathodes^60,61^.

## Conclusions

In summary, we can conclude that the infiltration process of Li_3_YCl_4_Br_2_ halide solid electrolyte into conventional lithium-ion battery cathode chemistries shows the following limitations:

- The dissolution and reprecipitation of the halide solid electrolyte using ethanol and DI water as solvents lead to a significant decrease in ionic conductivity, with a one-order-of-magnitude reduction compared to the pristine (untreated) state.
- The choice of solvents induces morphological and structural changes in the halide electrolyte.
- The maximum concentrations of halide solid electrolyte in ethanol (EtOH) and DI water are 30 wt% and 40 wt%, respectively.
- The concentration of EtOH and DI water influences the morphology and structure of the electrolyte, further compromising ionic conductivity and viscosity.
- The infiltration process results in the halide electrolyte primarily accumulating on the surface of the electrodes due to agglomeration, preventing effective penetration into the electrode pores.
- The halide electrolyte solution causes dissolution of NCM622 cathode components, leading to unwanted side reactions.
- The infiltrated NCM622 electrodes show significantly reduced electrochemical performance and increased resistance.

In general, these factors collectively explain the extremely low capacity observed in the infiltrated cells. The limited penetration of the recrystallized halide solid electrolyte, the formation of large agglomerates, and the reduced ionic conductivity restrict Li-ion transport within the NCM cathode. Moreover, the interfacial degradation between the halide electrolyte and NCM622 further increases resistance and reduces the electrochemically active area.

Therefore, we can conclude that possible pathways to improve performance include optimizing electrode porosity to facilitate deeper electrolyte penetration, tailoring solvent systems to minimize particle agglomeration and conductivity loss, and applying interfacial-stabilization strategies to suppress cathode dissolution and side reactions. These directions highlight the potential of infiltration-based processing while identifying the key challenges that must be addressed for practical implementation.

We can propose that, looking ahead, several practical directions could help improve the infiltration process and address the limitations identified in this study. (1) The choice of solvent remains a central parameter: non-polar systems preserve electrolyte integrity, whereas polar solvents enable dissolution but induce degradation. (2) Exploring mixed-solvent systems or tuning solvent polarity may help balance these effects. (3) Likewise, controlling the drying rate and temperature could reduce surface crystallization by limiting solvent-driven solute migration. (4) Surface-energy interactions between the electrolyte, solvent, and cathode framework also appear to play a decisive role, suggesting that surface-energy tuning, either through solvent selection, additives, or cathode surface treatments may support more uniform electrolyte distribution. (5) Reducing electrolyte particle size through nanoscale comminution or high-energy milling could further improve pore accessibility, particularly for non-polar dispersions. (6) Finally, alternative electrode fabrication routes, such as direct slurry-casting of NCM, conductive carbon, binder, and halide electrolyte, or hybrid infiltration slurry approaches, may offer more robust ionic percolation and improved interfacial contact. These strategies outline realistic pathways for advancing halide-based solid-state NCM cathodes with lithium metal anodes in future work.

## Supplementary Information

Below is the link to the electronic supplementary material.

Supplementary Material 1

## Data Availability

All additional data supporting the findings of this study are available from the corresponding author upon reasonable request.
