# Supplementary material for: Challenges of the infiltration method for halide-based solid‑state battery cathodes
Source: Sci Rep. 2026 Apr 5;16:16464. doi: 10.1038/s41598-026-47289-w (PMC13216298; doi:10.1038/s41598-026-47289-w)
Supplement: Supplementary file 1 — Supplementary Material 1 [file 41598_2026_47289_MOESM1_ESM.docx]

**Supporting Information**

**Experimental**

***Electrode preparation of PVDF/NMP-based****:* The poly-crystalline NCM622 cathode was prepared by blending obtained powder (LiNi_0.6_Co_0.2_Mn_0.2_O_2_ coated by LiNbO_3_ 1 wt%, NEI corp) (92 wt%), polyvinylidene fluoride (Sigma-Aldrich) binder (4 wt%) and carbon black (Super C65, Imerys) conductor (4 wt%) in N-methyl pyrrolidone (NMP, Sigma-Aldrich) via a centrifugal planetary mixing machine (THINKY MIXER, ARE-250) to obtain a uniformly mixed slurry and cast onto aluminum foil (Al, thickness = 15 μm) and dried at 120°C for 10 min to eliminate the NMP solvent. The NCM electrodes exhibiting 70% porosity were obtained as the best option for the infiltration process [S1]. The porosities of the electrodes were estimated by subtracting the summed volume of all pristine components from the volume of the prepared electrodes. The latter was determined by measuring the thickness of circular cut-outs (15 mm diameter) with a µm gauge. Finally, all electrodes were dried at 120°C for 16 h in a vacuum oven before transferring them into an Ar filled glovebox (H_2_O = < 0.1 ppm; O_2_ = < 0.1 ppm). Unless otherwise stated the infiltration process and all further handling were also conducted in an Ar-filled glovebox.

***Infiltration process of halide solution****:* The halide solid electrolyte powder (Li_3_YCl_4_Br_2_, denoted as LYCB) utilized in this study was kindly supplied as a developmental material by Saint‑Gobain Recherche, Paris (France). To prepare the solid electrolyte solution for the infiltration process, Li_3_YCl_4_Br_2_ was dissolved into ACN (acetonitrile, Sigma-Aldrich), ANI (anisole, Sigma-Aldrich), DBM (dibromoethane, Sigma-Aldrich), EtOH (ethanol, Sigma-Aldrich), HE (heptane, Sigma-Aldrich), HIB (hexyl isobutyrate, Sigma-Aldrich), TOL (toluene, Sigma-Aldrich), XYL (p-xylene, Sigma-Aldrich), and DI water solvents at 6, 11, 20, 30 and 40 wt%.

The volume of infiltration solution was adjusted to match the accessible pore volume of the electrode, calculated using 𝑉_pore_ = 𝜀 x 𝑉_electrode_, resulting in 𝑉_pore_ ≈ 0.30-0.35 mL per mL of NCM electrode. This ensured complete pore filling without oversaturation and enabled uniform distribution of the recrystallized electrolyte within the electrode structure.

To fabricate solid electrolyte infiltrated cathode electrodes were used the as-prepared coated NCM622-LiNbO_3_ composite electrodes were infiltrated by solid electrolyte solution into the pores of conventionally fabricated electrodes by immersion or dip coating processes. The infiltration route exploits the solubility of the Li_3_YCl_4_Br_2_ material in ethanol and recrystallization of the same after evaporation of the solvent at temperatures up to room temperature (RT) the following treatment at 150°C in a vacuum oven for 2 h a solid electrolyte/electrode is obtained, which can further be used in the same way as composite electrodes processed by conventional methods. A schematic of the infiltration route is shown in Figure 1.

***Porosity:*** The porosity of conventional electrodes before the infiltration route was measured on based the thickness of the composite electrode before and after densification via a roll-press where the porosity of as-prepared composite cathode samples is 70% [S1].

***Pellet density:*** The pellet density was determined by the geometry of the prepared pellet at 300 MPa and the respective weight of the pellet.

***Wettability:*** The wettability of as-prepared infiltrated cathode samples was measured based on photos obtained for 30 sec after dropping the Li_3_YCl_4_Br_2_-ethanol and Li_3_YCl_4_Br_2_-Di water solution on top of the cathodes as the best candidates among other solvents (Figure 6b and Figure S5).

***Ionic conductivity:*** The ionic conductivities of solid electrolyte powders without treatment, after treatment with various solvents and subsequent drying at 150°C for 2 h were measured by AC impedance spectroscopy. The powder samples were placed between two stainless steel rods (as blocking electrodes) within a polyetherether ketone (PEEK) liner/template with 16 mm diameter and pressed at 6 tons for 5 mins (300 MPa). Then the pressure was released to 0 MPa and the impedance was measured to investigate the pressure-dependence behavior of the conductivity. The ionic conductivity of the electrolytes was then determined using a specific equation from a previous work [S2]. The thickness of the pellet was around 1.0 mm depending on the amount of powder samples used. All preparation steps with samples were performed inside an Ar-filled glovebox. Electrochemical impedance spectroscopy (EIS) was performed at room temperature using a Gamry Interface 1010 E from 2 MHz to 10 Hz at a 10 mV amplitude in PEIS mode.

***Powder X-ray diffraction and Scanning electron microscopy:*** After infiltration, cathodes were dried under controlled conditions prior to analysis. X‑ray diffraction (XRD) was performed to identify changes in the crystalline structure of both the NCM622 cathode and the Li_3_YCl_4_Br_2_ electrolyte. The crystalline structure of the solid electrolyte powders and NCM cathodes infiltrated solid electrolyte was determined by X-ray diffraction (XRD) analysis using a PANalytical X’Pert Pro diffractometer in Bragg-Brentano geometry with Cu Kα radiation (45 kV, 40 mA) in a 2θ range of 5–80° at a scan rate of 0.03° s^−1^. The samples for the XRD measurements were prepared and sealed in an Ar-filled glove box using a sample holder equipped with a polymer cap. The polymer cap produces a broad scattering feature around 20-22° 2θ, which appears in all diffraction patterns and was not subtracted during background correction [S1].

The surface morphology (SEM, EDS and cross section) of the samples before and after the heating step was observed using field emission scanning electron microscopy (FE-SEM, ZEISS Supra 40) with energy-dispersive X-ray spectroscopy (EDS).

***Particle size distribution:*** Particle analyzer Litesizer 500 Anton-Paar Austria using Glass cuvettes, 24 °C. All solutions were sonicated for 10 min to minimize particle aggregation before measurement and equilibrated at the analyzer for 1 minute. The refractive index and viscosity parameters were selected according to each solvent used.

***Element analysis:*** ICP-OES measurements were performed to determine the Ni, Mn, Co, Li concentrations in the samples after immersing in ethanol solution with halide solid electrolyte were measurements using an inductively coupled plasma (ICP) optical emission spectrometry of PerkinElmer ICP–OES type OPTIMA 7300 DV. Inductively coupled plasma (ICP) analysis was conducted on the infiltration solutions to quantify elemental dissolution (Ni, Mn, Co, Li) from the NCM622 cathode into the solvent. For comparison, parallel tests were performed using deionized (DI) water as the solvent.

***Electrochemical testing of the infiltrated cathode in solid state cell configuration:*** For the electrochemical tests, the lithium metal anode (MTI, Li chips, diameter 15.6 mm, thickness 0.25 mm) was used as the counter electrode. The method used to prepare the lithium metal anode is described in [S3,S4]. To prepare the bilayers, 400 mg of electrolyte powder is placed in a Ø16 mm pellet die and pressed at 50 MPa for a few seconds. Then the infiltrated cathode (loading of 1 mAh cm^−2^ and about 8-9 mg cm^−2^) is placed on the solid electrolyte surface from one side and pressed at a pressure of 300 MPa and the pressure is maintained for 5 minutes. The lithium-metal anode was placed on the opposite side of the solid-electrolyte pellet, after which the assembled cell was mounted in the holder and a pressure of 10 MPa was applied during electrochemical measurements. A Gamry Interface 1010E with a current density of 0.05 mA cm^−2^ (C/20) in the potential range of 4.3 V and 2.7 V (vs Li/Li^+^) was used for the galvanostatic cycling with potential limiting measurements. Electrochemical impedance spectroscopy (EIS) was performed from 2 MHz to 10 Hz at 10 mV amplitude in PEIS mode.

**References:**

[S1] A. Tron, A. Paolella, A. Beutl, New insights of infiltration process of argyrodite Li_6_PS_5_Cl solid electrolyte into conventional lithium-ion electrodes for solid-state batteries, Batteries 9 (10) (2023) 503.

[S2] A. Tron, A. Orue, P. López-Aranguren, A. Beutl, Critical current density measurements of argyrodite Li_6_PS_5_Cl solid electrolyte at ambient pressure, J. Electrochem. Soc. 170 (2023), 100525.

[S3] A. Tron, R. Hamid, N. Zhang, A. Beutl, Rational optimization of cathode composites for sulfide-based all-solid-state batteries. Nanomaterials 13 (2023) 327.

[S4] A. Tron, A. Beutl, I. Mohammad, A. Paolella, Insights into the chemical and electrochemical behavior of halide and sulfide electrolytes in all-solid-state batteries, Energy Advances (2025).


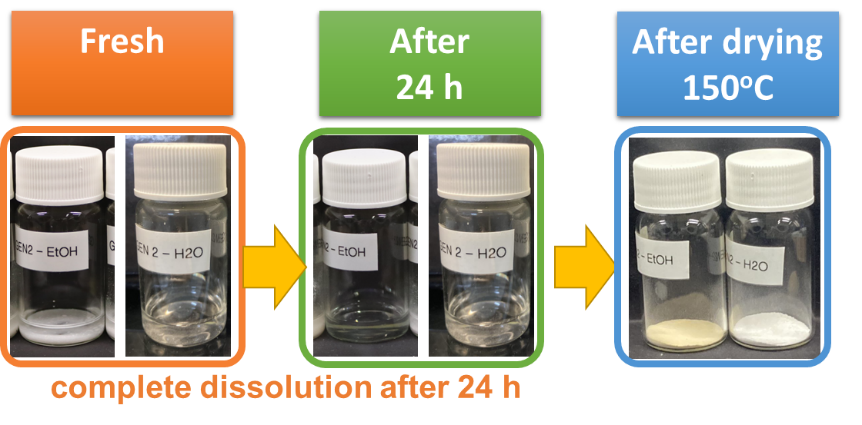


Figure S1. *Photographs of vials with Li_3_YCl_4_Br_2_ treated with the solvents EtOH and DI water with a loading of 10 wt% Li_3_YCl_4_Br_2_ before and after 24 h treatment and after drying at 150^o^C in vacuum. The EtOH and DI water completely dissolve Li_3_YCl_4_Br_2_.*

Table S1. *Solvents and their compatibility with the Li_3_YCl_4_Br_2_ solid electrolyte indicated by changes in their XRD pattern, surface morphology (SEM), ionic conductivities and/or density.*

| **Solvents** | **Changes observed in XRD and SEM** | **Ionic conductivity / S cm^−1^ @ 100 MPa** | **Density / g cm^−3^** |
| --- | --- | --- | --- |
| Pristine solid electolyte | - | 8.38 10^−4^ | 2.42 |
| DI water (DI H_2_O) | Yes | 2.64 10^−4^ | 1.85 |
| Ethanol (EtOH) | Yes | 4.22 10^−4^ | 2.15 |
| Toluene (TOL) | Yes | 8.49 10^−4^ | 1.93 |
| p-Xylene (XYL) | Yes | 5.94 10^−4^ | 1.99 |

*Note: The "Changes Yes/No" column has been expanded to specify the type of XRD modification observed (peak broadening, intensity variation, or appearance of minor reflections). No significant peak-position shifts were detected within the resolution of the instrument. The observed broadening and intensity changes are attributed to surface reactions and partial crystallization of the halide electrolyte rather than bulk structural changes of the NCM cathode.*


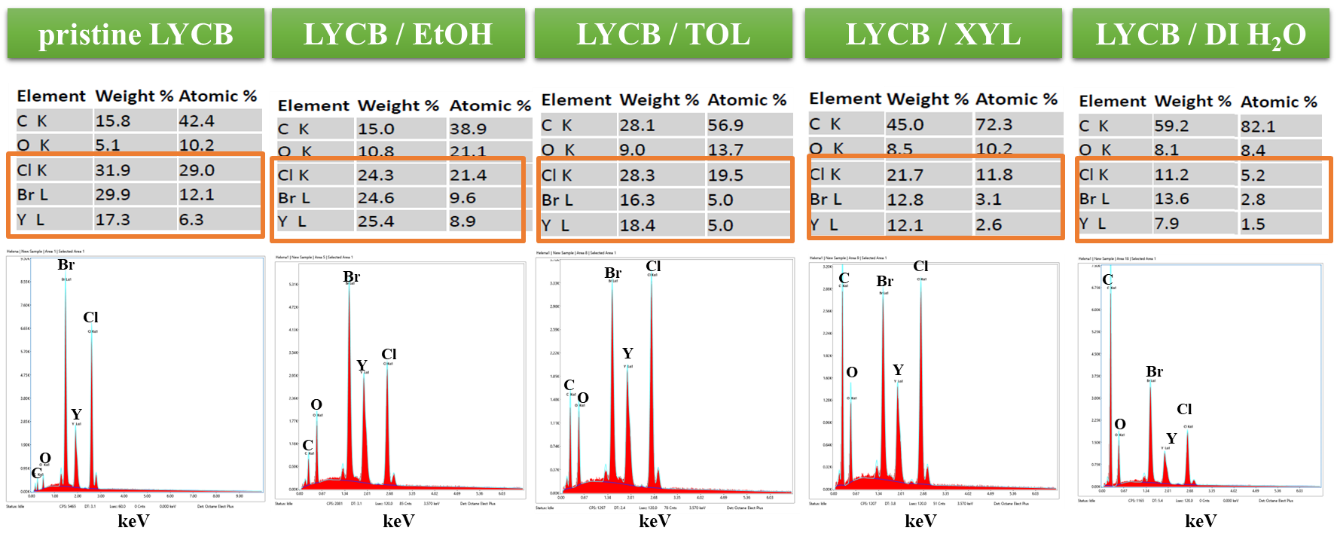


Figure S2. *EDS analysis of Li_3_YCl_4_Br_2_ (LYCB): pristine and treated with the solvents EtOH, TOL, XYL, and DI water after drying at 150^o^C in vacuum.*


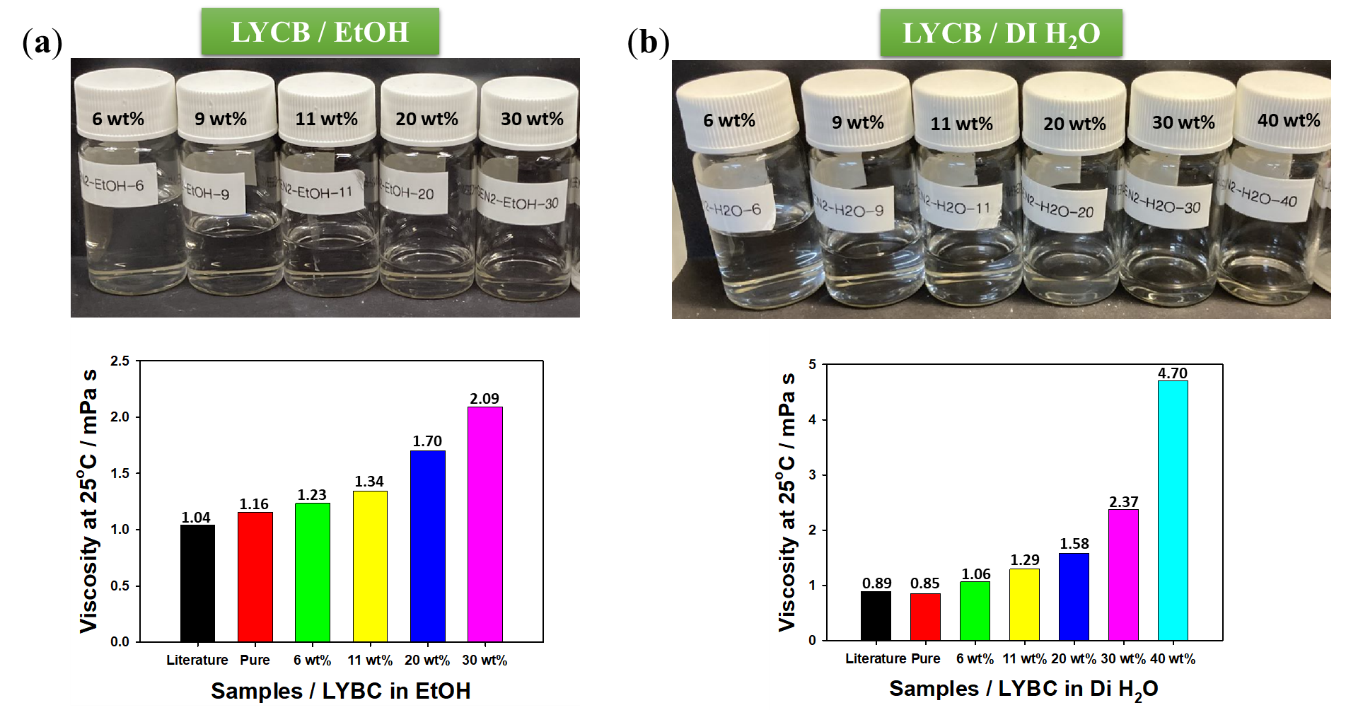


Figure S3. *Viscosity of Li_3_YCl_4_Br_2_ electrolyte into the solvents (a) EtOH and (b) DI water.*


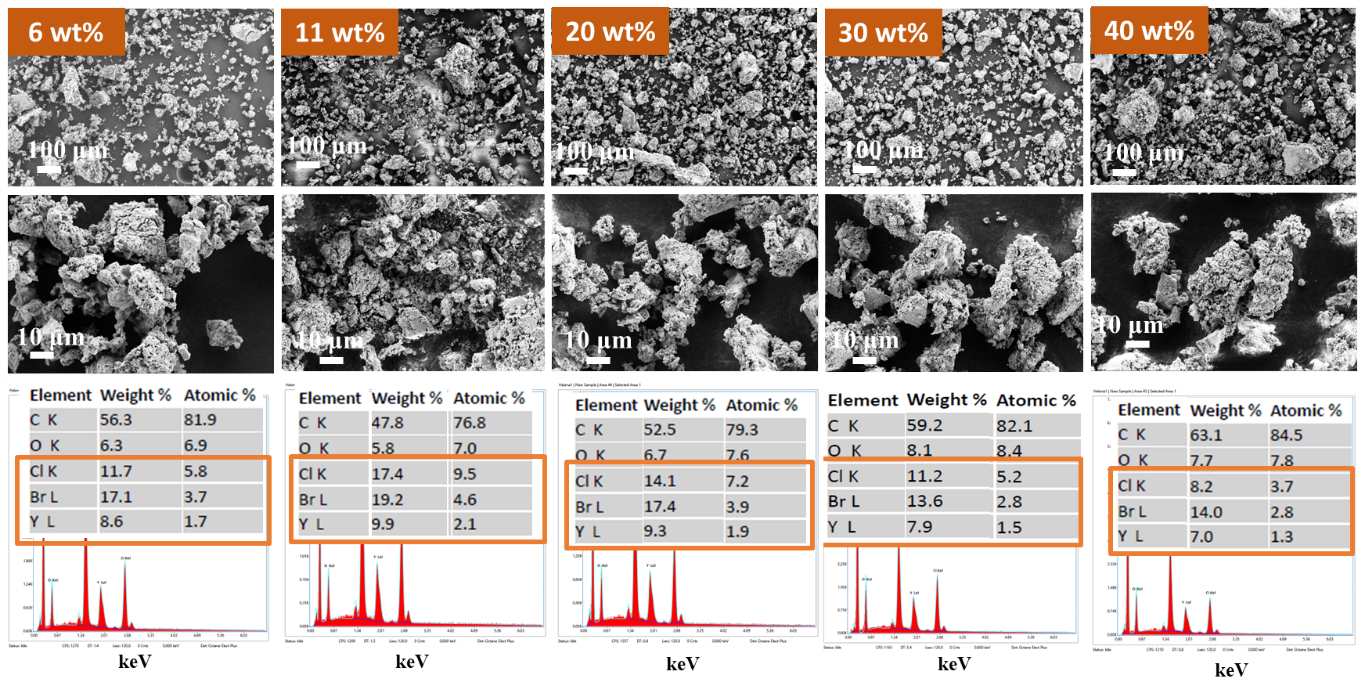


Figure S4. *SEM images and EDS analysis of Li_3_YCl_4_Br_2_ treated with the solvents DI H_2_O water with a loading of 6, 9, 11, 20, 30 and 40 wt% after drying at 150^o^C in vacuum.*

Table S2. *Solvents and their compatibility with the Li_3_YCl_4_Br_2_ solid electrolyte indicated by changes in their XRD pattern, surface morphology (SEM), ionic conductivities and/or density.*

| **Solvents** | **Changes observed in XRD or SEM** | | **Ionic conductivity / S cm^−1^ @ 100 MPa** | | **Density / g cm^−3^** | | **Vicsosity / mPa sec** | |
| --- | --- | --- | --- | --- | --- | --- | --- | --- |
|  | Ethanol  (EtOH) | Di water  (DI H_2_O) | Ethanol  (EtOH) | Di water  (DI H_2_O) | Ethanol  (EtOH) | Di water  (DI H_2_O) | Ethanol  (EtOH) | Di water  (DI H_2_O) |
| 0 wt% (pristine solid electolyte) | - | - | 9.78 10^−4^ | - | 2.42 | - | 1.04 | 0.855 |
| 6 wt% | Yes | Yes | 3.48 10^−4^ | 2.11 10^−4^ | 2.49 | 2.55 | 1.2338 | 1.062 |
| 11 wt% | Yes | Yes | 3.66 10^−4^ | 3.53 10^−4^ | 2.55 | 2.47 | 1.3436 | 1.2924 |
| 20 wt% | Yes | Yes | 4.67 10^−4^ | 4.41 10^−4^ | 2.57 | 2.56 | 1.7004 | 1.5782 |
| 30 wt% | Yes | Yes | 4.72 10^−4^ | 4.44 10^−4^ | 2.55 | 2.49 | 2.0918 | 2.374 |
| 40 wt% |  |  |  | 4.51 10^−4^ |  | 2.52 |  | 4.7034 |

*Note: The "Changes Yes/No" column has been expanded to specify the type of XRD modification observed (peak broadening, intensity variation, or appearance of minor reflections). No significant peak-position shifts were detected within the resolution of the instrument. The observed broadening and intensity changes are attributed to surface reactions and partial crystallization of the halide electrolyte rather than bulk structural changes of the NCM cathode.*


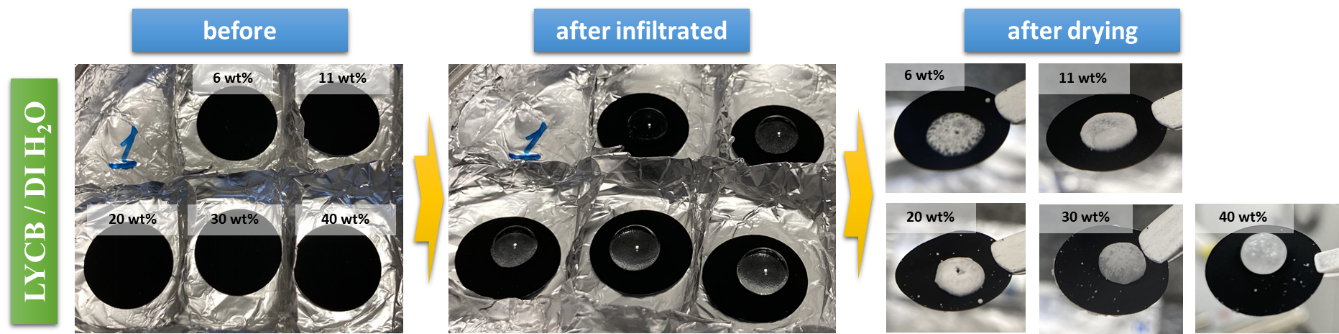


Figure S5. *Photographs of conventional NCM622 cathodes (CAM:CA:Binder / PVDF+NMP) before and after infiltration process via Li_3_YCl_4_Br_2_ into the solvents DI H_2_O water with a loading of 6, 9, 11, 20, 30 and 40 wt% and after drying at 150^o^C in a vacuum. Note: As shown in Figure S5, the Li_3_YCl_4_Br_2_-solvent droplets do not infiltrate the porous structure of the conventional NCM622 cathodes. The high surface energy of these electrodes leads to poor wetting, causing the droplets to remain on the surface and crystallize during drying, even at electrolyte loadings from 6 to 40 wt%. This behavior confirms that the observed deposits originate from surface crystallization rather than true infiltration, and explains why quantitative contact-angle measurements are not representative under these conditions.*

Table S3. *Particle size analyzing of Li_3_YCl_4_Br_2_ electrolyte into the solvents EtOH, TOL, XYL, and DI water obtained from DLS analysis and SEM images (Figure 3).*


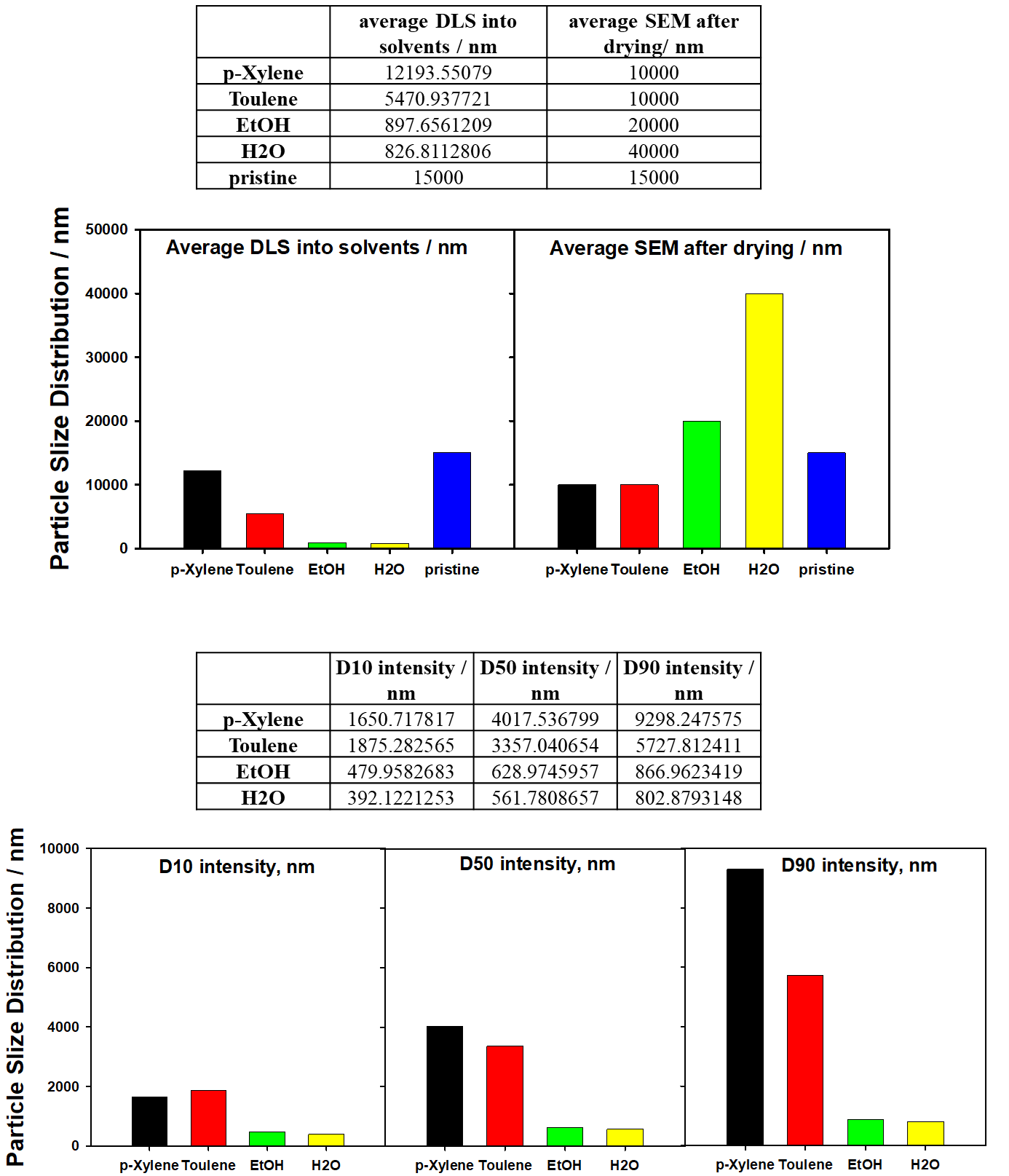


Table S4. *Particle size analyzing of Li_3_YCl_4_Br_2_ electrolyte into the solvents EtOH, TOL, XYL, and DI water obtained from DLS analysis and SEM images after dissolution for 24 h (Figure 3).*


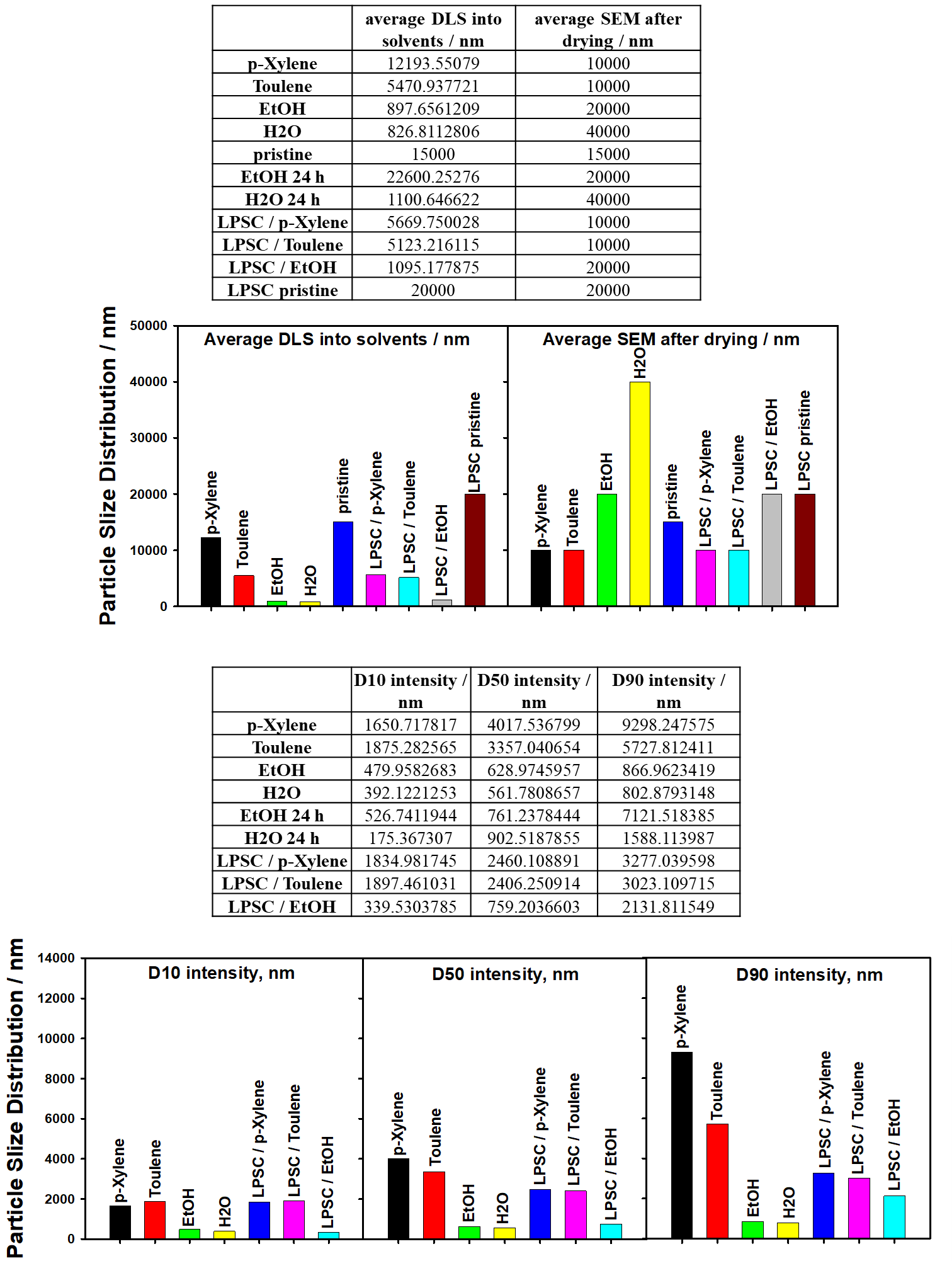


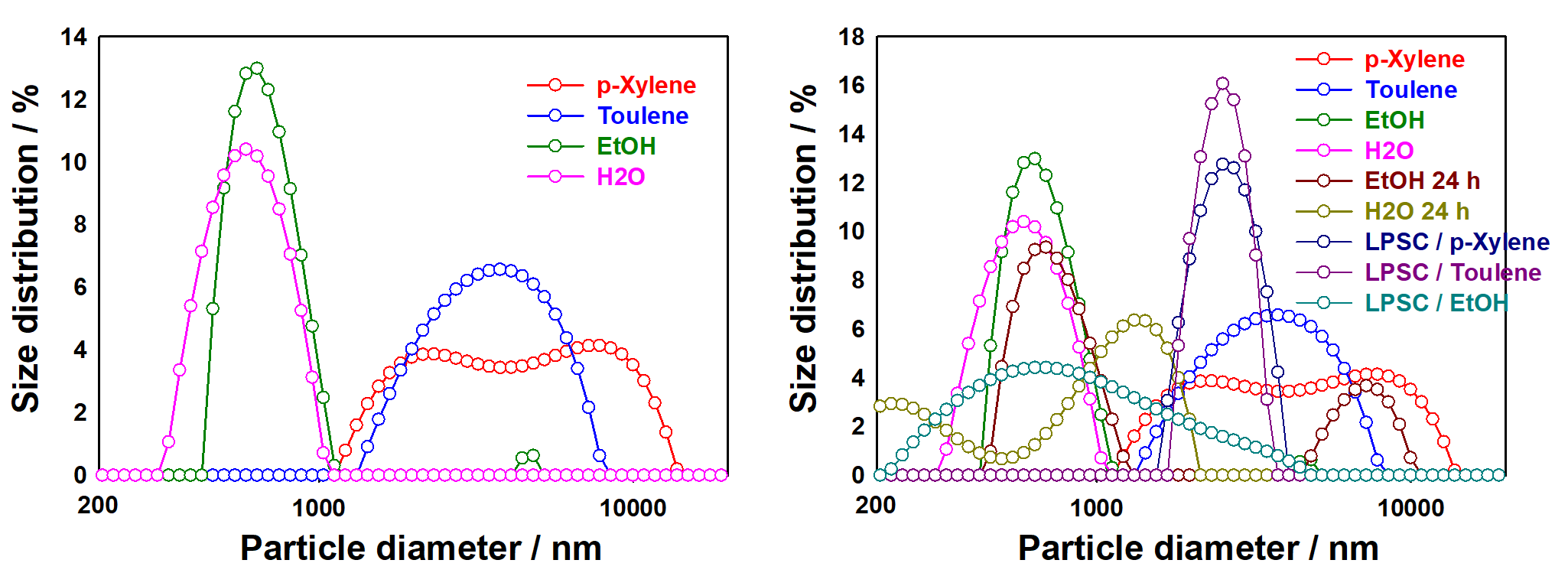


Figure S6. *Particle size analyzing of Li_3_YCl_4_Br_2_ electrolyte into the solvents EtOH, TOL, XYL, and DI water obtained from DLS analysis and SEM images after dissolution for 24 h (Figure 3).*


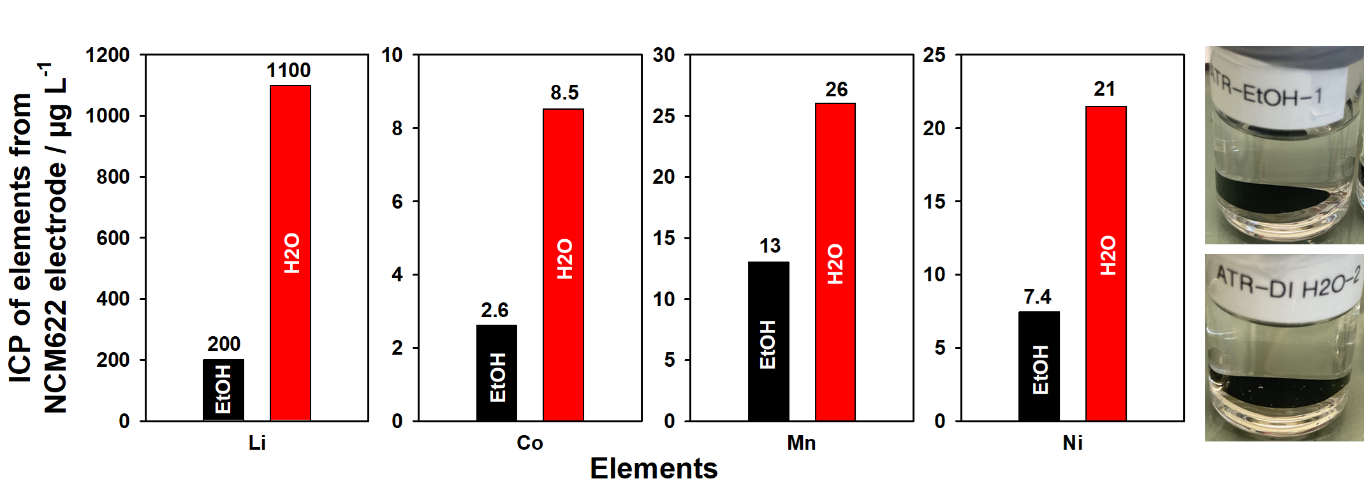


Figure S7. *ICP of Li_3_YCl_4_Br_2_ solution of conventional NCM622 electrodes into EtOH and DI H_2_O water.*


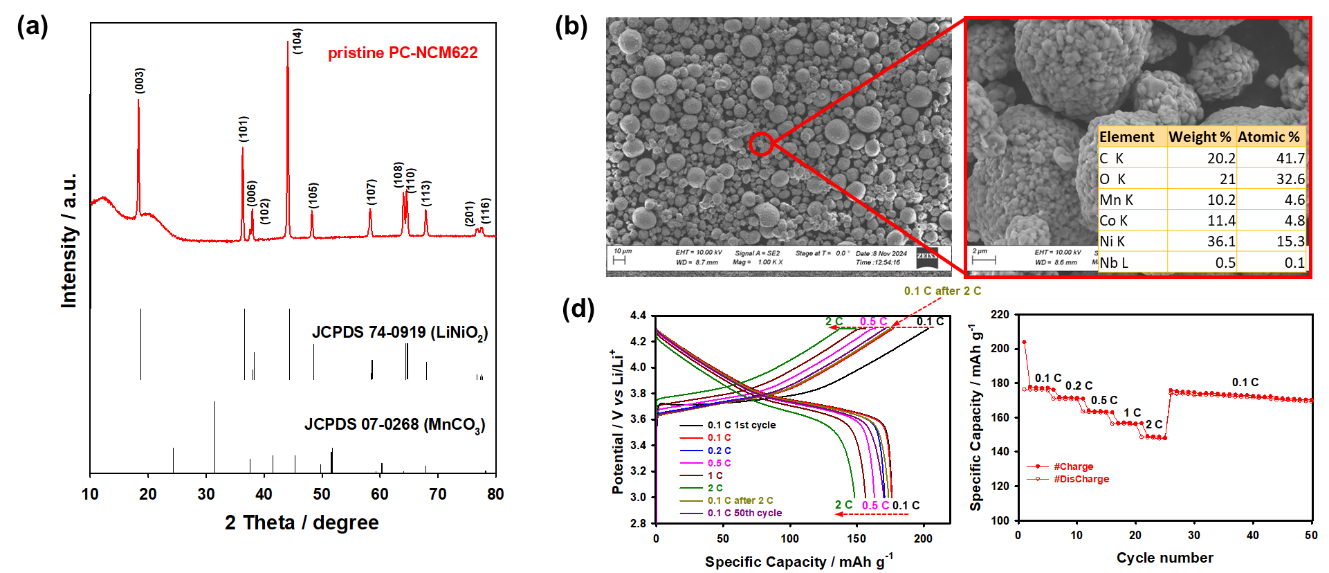


Figure S8. *(a) XRD pattern and (b) SEM and XRF of the PC-NCM622 coated via LiNbO_3_ powder, (c) Rate capabilities and (d) voltage curves of the PC-NCM622 coated LiNbO_3_ electrode tested into the liquid electrolyte of 1 M LiPF_6_ in EC DEC (1:1 v/v) in the potential range of 3.0−4.3 V (vs. Li/Li^+^) at various current densities at room temperature.*

Table S5. *Overview of characteristics and wettability of NCM electrodes (Li_3_YCl_4_Br_2_ solution into EtOH) used for infiltration route.*

| **Concentration of EtOH** | **pristine** | **6 wt%** | **11 wt%** | **20 wt%** | **30 wt%** |
| --- | --- | --- | --- | --- | --- |
| Mass electrode before infiltration / mg | 15.6 | 15.8 | 16.2 | 16.7 | 17.3 |
| Mass electrode after infiltration / mg | 15.6 | 16.7 | 18.9 | 20.1 | 27.6 |
| Loading of SE / mg cm^-2^ | - | 0.5 | 1.5 | 1.9 | 5.8 |
| Thickness electrode before infiltration / μm | 40 | 41 | 41 | 41 | 42 |
| Thickness electrode after infiltration / μm | 40 | 76 | 106 | 155 | 217 |
| Wettability with electrolyte solution | - | High | High | High | High |
| Viscosity | Low | Low medium | Medium | High medium | High |
| Particle size (SEM) / μm | 10…20 | | | | |
| Particle size (DLS) / μm | ~1 | | | | |
| Pores of NCM electrode / μm | Less than 1 | | | | |
| Structure changes | No | Yes | Yes | Yes | Yes |
| Morphology changes | No | Yes | Yes | Yes | Yes |
